# Methylome-wide association study of whole blood DNA in the Norfolk Island isolate identifies robust loci associated with age

**DOI:** 10.18632/aging.101187

**Published:** 2017-02-28

**Authors:** Miles C. Benton, Heidi G. Sutherland, Donia Macartney-Coxson, Larisa M. Haupt, Rodney A. Lea, Lyn R. Griffiths

**Affiliations:** ^1^ Genomics Research Centre, Institute of Health and Biomedical Innovation, School of Biomedical Sciences, Queensland University of Technology, Kelvin Grove, Queensland, 4059, Australia; ^2^ Kenepuru Science Centre, Institute of Environmental Science and Research, Wellington 5240, New Zealand

**Keywords:** aging, DNA methylation, epigenetics, GLMnet, Norfolk Island

## Abstract

Epigenetic regulation of various genomic functions, including gene expression, provide mechanisms whereby an organism can dynamically respond to changes in its environment and modify gene expression accordingly. One epigenetic mechanism implicated in human aging and age-related disorders is DNA methylation. Isolated populations such as Norfolk Island (NI) should be advantageous for the identification of epigenetic factors related to aging due to reduced genetic and environmental variation. Here we conducted a methylome-wide association study of age using whole blood DNA in 24 healthy female individuals from the NI genetic isolate (aged 24-47 years). We analysed 450K methylation array data using a machine learning approach (GLMnet) to identify age-associated CpGs. We identified 497 CpG sites, mapping to 422 genes, associated with age, with 11 sites previously associated with age. The strongest associations identified were for a single CpG site in *MYOF* and an extended region within the promoter of *DDO*. These hits were validated in curated public data from 2316 blood samples (MARMAL-AID). This study is the first to report robust age associations for *MYOF* and *DDO*, both of which have plausible functional roles in aging. This study also illustrates the value of genetic isolates to reveal new associations with epigenome-level data.

## INTRODUCTION

Epigenetics is a rapidly developing area of research and refers to the heritable, but reversible, regulation of various genomic functions including gene expression. This provides mechanisms whereby an organism can dynamically respond to a change in its environment and modulate gene expression accordingly. As such, epigenetic mechanisms can have a profound effect on phenotype, including disease risk and progression. One such epigenetic mechanism is DNA methylation, with cytosine residue methylation at CpG dinucleotides well documented in many organisms. The investigation of an individual's methylation pattern can reveal a lifetime record of environmental exposures as well as potential disease specific patterns.

Aging in humans has been associated with marked remodelling of the epigenetic architecture in terms of DNA methylation patterns [1–3]. To date these studies have identified age-related CpG associations in healthy populations [4,5], and age-related CpGs associated with disease susceptibility [6]. Studies have assessed the extremes of the age distribution by comparing the methylomes of centenarians with newborns [7,8]. One study proposed the idea of a methylation 'clock' for tissue specific aging [9], while another demonstrated the use of DNA methylation to predict all-cause mortality in later life [10]. This breadth of evidence highlights that age-associated methylation variants could be important in influencing age-related disease.

Most studies of methylation in relation to aging have focused on the use of whole blood DNA given its relative accessibility when compared to other cell and tissue types. The Infinium Human Methylation 450 Beadchip (450K Array) has been the most popular technology for conducting such methylome-wide association studies (MWAS). A large number of MWASs focusing on blood have been conducted and data for >4700 individual samples have been posited in the public repository called MARMAL-AID [11]. Interestingly, a recent meta-analysis of whole blood MWASs applied a new bioinformatics method to identify differentially methylated regions (DMRs) associated with aging in humans [12]. Briefly, CpG sites were identified based on their genomic features, defining groups of adjacent sites based on their density, and applying either a single site or region centric analysis. The authors suggested that this design should allow for better comparison across other studies of the similar design. This study resulted in a robust list of DMRs that vary across the lifespan and may also have potential importance in aging biology [12]. More recently, a review by Jones et al., [1] also provided a meta-analysis of age-related methylation papers and explored the overlap between the most significant CpG sites across 7 separate studies to identify 11 CpG sites commonly associated with age [1].

Most age-based MWAS conducted to date have been performed in unrelated cohorts collected from general populations. Whilst such studies can offer the advantage of large cohort sizes they can be negatively affected by confounding factors such as underlying genetic substructure and highly variable environmental influences. Genetically isolated populations may help overcome such issues due to having a reduced genomic and environmental diversity when compared to general populations [13]. The Norfolk Island (NI) population is a genetic isolate with a well-documented history [14]. Norfolk Island is geographically remote, located ~1600kms off the East Coast of Australia. The modern NI population was originally founded in the late 1780's on Pitcairn Island by 9 Bounty Mutineers and 6 Polynesian wives and in 1856 the founder descendants relocated to NI [15]. Given its remoteness, the population grew in almost complete isolation from mainland Australia. This, along with the small island size and strict immigration policies, has ensured that both genetic and environmental conditions shared by all NI individuals have remained fairly homogeneous. To date the isolate has been well characterised genetically and phenotypically as part of the Norfolk Island Health Study (NIHS) [16].

In an effort to further our understanding of age-related methylation we conducted a MWAS of whole blood DNA in a healthy cohort from the isolated population of NI. We applied a statistical algorithm called GLMnet [17], a machine-learning approach which conducts simultaneous analysis of CpGs by allowing mixing of ridge regression and Lasso (least absolute shrinkage and selection operator) in an elastic-net framework. We validated our top findings in the large cohort of publicly available methylation data from MARMAL-AID.

## RESULTS

### NI aging and methylation

DNA extracted from blood samples from 24 healthy females from the well documented NI genetic isolate [16,18] were assayed for methylation levels at probes across the genome using Infinium Human Methylation 450 Beadchips (450K Arrays). Individuals ranged in age from 24 to 47 years, with a mean age of 36 years. We explored associations between age and methylation (beta-value) using a GLMnet (mixture of lasso and ridge regression) approach, and identified a total of 497 CpG sites associated with age (Additional File: Table S1). We then compared the 422 genes to which the 497 CpG sites mapped to those identified in a recent meta-analysis [12]. We observed 5 genes from our study which were consistent with the meta-analysis by Bacalini et al., (2015): *ABCC4*; *CSNK1D*; *EDARADD*; *ELOVL2*, and *OTUD7A*. Interestingly, we found numerous other age-related CpG sites that had not been previously identified.

**Figure 1:**
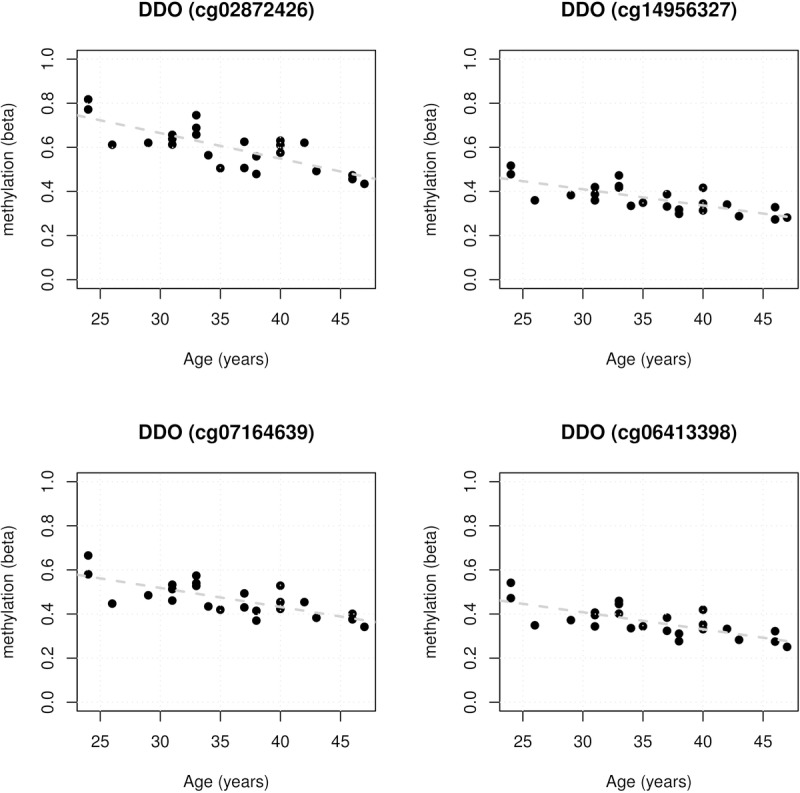
Four promoter associated *DDO* CpG age-associations for the 24 healthy female Norfolk Island samples showing statistically significant reduction in methylation with age. Regression statistics are displayed within each panel.

As this is a multi-marker approach there is no individual p-value associated with each CpG site as there would be under a more traditional single-marker test. Using our approach, we applied the absolute range in methylation as a form of ranking, under the assumption that the ‘larger absolute range’ loci would have more biological relevance (Additional File: Table S1). Using this approach of CpG sites and mapping to annotated genes, cg02872426 mapping within the transcription start site (TSS200) of *DDO* showed the largest difference in methylation. Strikingly, further analysis revealed a differentially methylated region (DMR) encompassing 4 CpG sites in the promoter region of *DDO* (OMIM:124450): cg02872426; cg14956327; cg07164639, and cg06413398 (Figure 1). We then utilised available public data as an “independent cohort” to further explore these sites.

### Validation in public data

**Figure 2:**
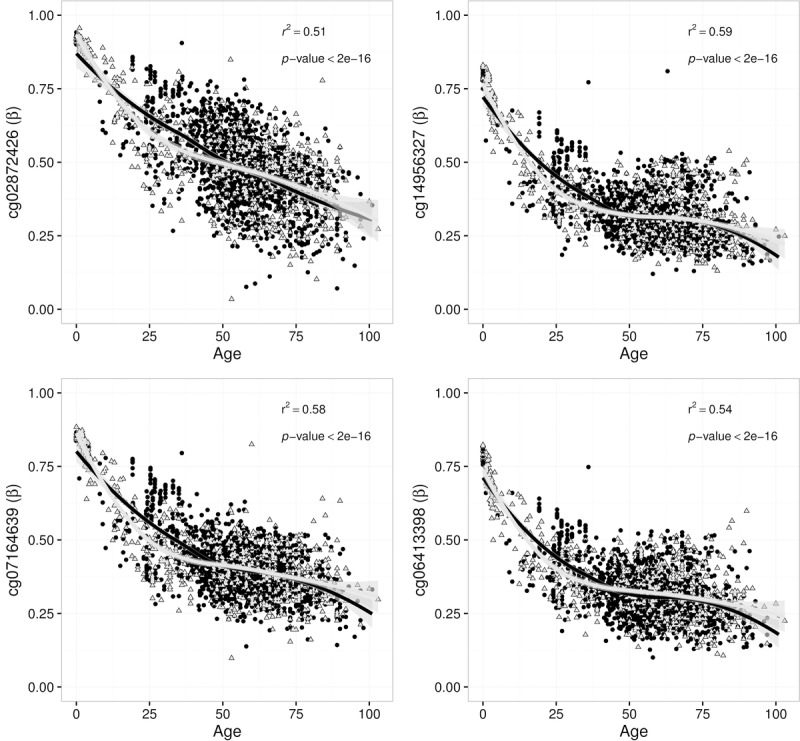
Four *DDO* promoter CpG sites associated with human age in white blood cells from 2316 samples sourced from the MARMAL-AID methylation repository. Each association is fitted with an overall loess regression model, with the regression statistics shown in the top right of each panel. Points are coloured and shaped to represent both males (black, circles) and females (grey, triangles) separately.

**Figure 3:**
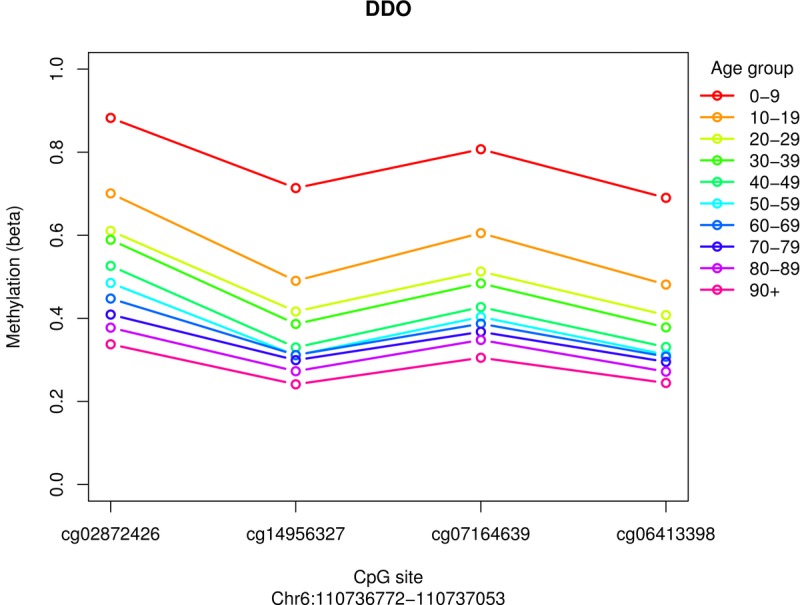
Public blood data age categorised for 4 promoter associated *DDO* probes, portrayed in the same fashion as demonstrated in Bacalini et al., 2015. Mean methylation values in 10 age classes are reported for each CpG probe within the *DDO* promoter.

At the time of analysis there were >4700 blood samples listed in MARMAL-AID [11], 2316 of which had age recorded (range 0-103 years, mean age 54 years). These data were obtained and all *DDO* CpG sites were extracted. We identified associations between *DDO* methylation and age in the public data; the methylation spectrum ranged from ~100% in fetal blood samples through to <25% in samples from individuals >75 years of age (Figure 2). Furthermore, although we had only assayed females in the NI sample, the age-dependent association of *DDO* promoter methylation was apparent in both males and females in the public data set. Using the same visualisation methods described in the meta-analysis performed by Bacalini et al., we observed consistent hypomethylation in the older age categories (Figure 3). In addition, we explored age associations across all *DDO* probes which are present on the Illumina 450K array (Figure S1.). Interestingly, a fifth CpG site, cg02872426 (also in the promoter region), showed strong correlation with age in the public data set. Overall, this is the first observation of a robust 5 CpG site DMR at the promoter region of *DDO*.

**Figure 4:**
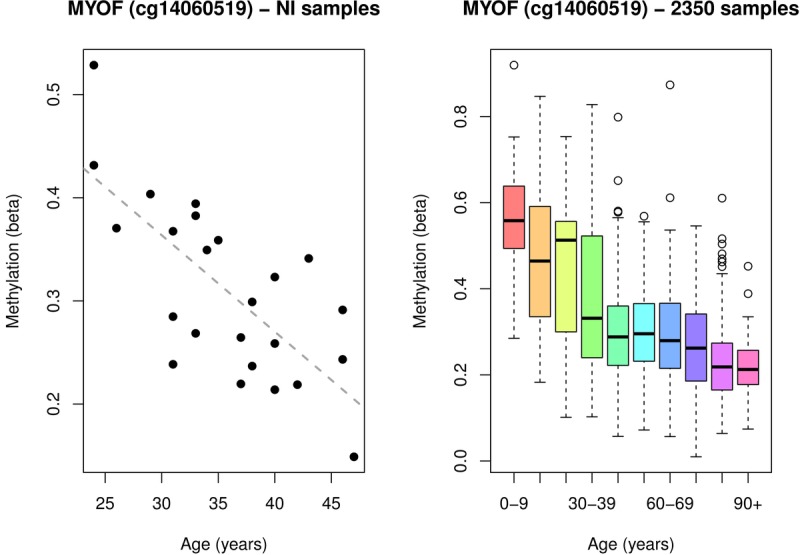
A single *MYOF* CpG site associated with age in: (**A**) the 24 healthy female Norfolk Island samples, and (**B**) 2316 public blood samples sourced from MARMAL-AID.

The next top hit after *DDO* when ranking the results by absolute range was cg14060519, located in the gene body of *MYOF* (Additional File: Table S1). This CpG was also validated in the public data (Figure 4), showing a decrease in methylation with age inde-pendent of sex. Interestingly *MYOF* (OMIM:604603) has been recently associated with aging in a comparison of RNASeq and 450K methylation data [19]. However, this paper identified a different CpG site, cg14428166, as their top mediator. The site identified in that study (cg14428166) is 99Kb away from the site associated in the NI cohort and validated here in the public blood data (cg14060519). Additionally, we were unable to replicate this association between age and cg14428166 in either the NI cohort or public methylation data.

### Biological significance of methylation with aging

The 497 age-related CpG sites identified in this study map to 422 unique genes. We noted overlap of 5 of these genes with those from Bacalini et al., [12]. To further investigate the biological context of these genes we explored the overlap with the 305 genes listed in the GenAge Database of Ageing-Related Genes [20]. We identified a total of 11 genes in common between the 2 gene sets: *STAT3*, *TNF*, *IGF2*, *POLA1*, *DGAT1*, *HSF1*, *EPS8*, *HIF1A*, *NFKBIA*, *GCLM*, and *NGFR*.

We examined our CpG sites against those reported to define the epigenetic clock model recently detailed in [9]. Of the 353 CpG sites used in the epigenetic clock we observed an overlap of 3: cg09809672, cg19761273 and cg07849904 with our panel. In addition, from the 344 unique genes (representing the aforementioned 353 CpG sites) we identified 8 as overlapping with our findings: *SYNE1*, *LGALS1*, *SLCO3A1*, *EDARADD*, *CSNK1D*, *KLF14*, *TBX5*, *MN1*, and *SCD5*.

A recent Nature Communications meta-analysis explored the interactions of age, gene expression and DNA methylation in whole blood [19]. Peters et al., reported 1497 genes as showing age associated expression. When we compared these with our data we observed an overlap of 41 genes between our gene list and the 1497 of Peters et al. Interestingly, one of these overlapping genes was *MYOF*, whereby they identified a significant association with age and gene expression, attributed to be methylation at CpG site cg14428166, located ~ 99kb away from the CpG site identified in this study (cg14060519).

More recently Jones et al., reported results of a meta-analysis of previously published experimental data [1]. Their final selection of a robust panel of common age-related markers identified 11 CpG sites (and genes) consistent across at least 4/7 studies. Our analysis also identified 2/11 of these genes and their respective CpG sites, *EDARADD* (cg09809672) and *GREM1* (cg21296230).

### Functional enrichment of genes with age-related methylation

To further explore the potential biological roles of the 422 genes represented by the 497 aged associated CpG sites we performed a functional enrichment analysis using the ToppGene Suite [21]. The GO Biological Processes functional category provided significant enrichment in 27 pathways passing a Bonferroni corrected threshold (Additional File: Table S2). Interestingly these pathways included numerous biological processes pertinent to aging, including: positive regulation of RNA metabolic process (p=0.001); muscle structure development (p=0.003); long-chain fatty-acyl-CoA metabolic process (p=0.01), and neurogenesis (p=0.01).

As we had access to multiple layers of genomic data for the NI population we were also able to examine the variance of *DDO* and *MYOF* gene expression in the NI cohort. Norfolk Island expression data was collected using Illumina HT-12 arrays for 330 of the NI individuals [18] with an age range of between 18-86 years within this sample group. When exploring age and methylation interactions we observed no correlation between expression of either *DDO* (Pearsons r 0.04, p-value 0.73) or *MYOF* (Pearsons r 0.02, p-value 0.89) transcripts present on the array. Additionally, we observed no association between transcript and methylation at the *DDO* promoter CpG sites (cg07164639 r=0.16 p=0.14; cg00804078 r=0.16 p=0.17; cg06413398 r=0.17 p=0.14; cg02872426 r=0.14 p=0.22; cg20011134 r=0.02 p=0.86).

## DISCUSSION

Here we report MWAS of peripheral blood DNA methylation in the NI genetic isolate. It is well established that genetic isolates offer advantage to the study of complex traits and disorders at a genomic level [13], but few studies have yet to explore this in the context of epigenetics [22,23]. Along with genetic homogeneity, one of the major benefits of studying an isolated population is the potential for reduced environmental effects, i.e. a shared environment (diet, weather, temperature, social structure). Using a cohort of healthy females from the NI population we identified numerous age-related methylation sites, many of which confirm already published research. Interestingly, several CpGs were uniquely identified in our study and validated in a large Illumina 450K public data set demonstrating the utility of population isolates in epigenetic association studies. Not only does this demonstrate the potential power of genetic isolates, but also the reliability and reproducibility of Illumina 450K DNA methylation data. Evidence for the reliability of Illumina 450K data has been presented in numerous studies, including our own [24], in particular accurate and precise estimates of methylation down to 2-4% detection levels [25–28] can be achieved. In this study in-silico validation was performed to confirm our findings using over 2300 individuals from 14 independent studies. This weight of evidence provides confidence that the findings in our study are valid and not due to any technical errors associated with the 450K arrays.

Two genes identified in our study were *DDO*, containing a set of 5 CpG sites forming a robust DMR across the promoter region, and *MYOF* with a single CpG site in the gene. Methylation of specific CpG sites in these two genes is reported here for the first time to be associated with age in a healthy human cohort.

Previously, association of methylation at a single CpG *DDO* site (cg14956327) with age has been reported in a cohort of northern Europeans, however this study concentrated on older individuals at risk of metabolic syndrome [2]. In contrast our study subjects were selected to be as healthy as possible (never smoked, minimal metabolic risk factors), and were of an overall younger age (<47 years old). Our study initially identified four *DDO* promoter CpG sites as age-associated in this healthy female NI cohort. We then validated this by using publicly available data for 2316 blood samples from mixed sex subjects, confirming the association and identifying a fifth age-associated *DDO* CpG site also in the promoter region (see Additional Figure S2). As such our results confirm that the age-association identified across *DDO* promoter CpG sites is observed in both female and male individuals from all age ranges. Moreover, it is interesting to note that *DDO* was not identified in the majority of previous studies, which leads us to suggest that the machine learning method we have employed here at least offers additional value to existing analytical methods, and should be used to complement and enhance such approaches.

D-aspartate oxidase is an enzyme encoded by the *DDO* gene. The protein, a peroxisomal flavoprotein, catalyses the oxidative deamination of D-aspartate (D-Asp) and N-methyl D-aspartate (NMDA) [29]. The current body of literature detailing the role of DDO suggests that it is crucial to several metabolic processes, many of which involve the transfer of electrons and generation of reactive oxygen species [30]. Biologically the impact of these processes over time is known to result in an accumulation of these potentially damaging components, as in the situation of reactive oxygen species generated by the mitochondria. One theory is that the accumulation of these free-radicals over time is responsible for increased damage to important cellular components, which may contribute directly to the aging process. It has been reported that *DDO* expression is highest in the brain of both animal models and humans [31,32]; it is nearly absent during embryonic and perinatal development and progressively increases during adulthood. Recently, a murine study reported that *DDO* promoter demethylation enables postnatal *DDO* expression, and that constitutively suppressed *DDO* expression (in *DDO* knock-out mice) leads to increased extracellular D-Asp levels in the brain, resulting in precocious neuronal cell death triggered by excessive NMDA receptor stimulation [33]. This suggests a key role for DDO in preventing neurodegeneration during brain aging. Our results suggest this may also be the case in humans. We observed demethylation in blood and it continues throughout life, while in the Punzo et al. study, conducted using whole mouse brain, the demethylation appeared to plateau to a level of approximately 30% at 3 weeks post partum (around time of weaning).

We also identified DNA methylation at cg14060519 in *MYOF* as being associated with age in the NI cohort. Methylation of this CpG has not been previously associated with age, however another CpG in an exon of *MYOF* (cg14428166), which is ~99Kb away from cg14060519, has been previously associated with aging in a very large meta-analysis exploring interactions between age, gene expression (RNAseq), and methylation (450K) [19]. Of note, we were able to identify differential methylation of a previously unreported age-associated CpG site in *MYOF* in a relatively modest sample size from a genetic isolate with this observation validated strongly in public data (Figure 4). Further investigation of the genomic region around cg14060519 revealed no other CpG's within +/−2kb of this position. However, the intronic location of this CpG is within an annotated regulatory hotspot, featuring DNase I hypersensitivity, an H3K27Ac mark and ENCODE CHIP-seq data. *MYOF* codes for a protein called myoferlin [34]. Myoferlin is very similar structurally to dysferlin, both belong to the ferlin family of proteins. These are calcium-sensing, membrane-associated proteins which play an important role in muscle membrane repair and growth [35]. Mutations in these ferlin proteins can cause muscle weakness affecting both proximal and distal muscles. Myoferlin has been suggested as a candidate gene and potential modifier for muscular dystrophy [34], and is required for insulin-like growth factor response and muscle growth [36]. Furthermore, myoferlin has also been shown to be highly expressed in endothelial and vascular tissues where it has a role in membrane integrity via its regulation of vascular endothelial growth factor (VEGF) receptor-2 stability and signalling [37]. These data suggests a plausible biological relevance of a role for *MYOF* in the aging process.

While differentially methylated regions (multiple CpG sites) are most commonly reported, there is evidence that single-marker CpG sites, such as the one we identified in *MYOF*, can show associations with traits of interest, and indeed, functionality. Nile et al., (2008), reported that methylation status at a single CpG site in the promoter of IL6 affected the regulation of gene expression of IL6, potentially influencing rheumatoid arthritis [38]. In another study, by Fürst et al., (2012), a single differentially methylated CpG was identified to affect transcription of ESR1 [39]. More recently, Dick et al., (2014) identified a single CpG site from whole blood associated with BMI in obese individuals [40]. They also identified the same correlation at the same single CpG site in adipose tissue, providing an excellent example of a robust single marker association and its translation to a potential biomarker.

DNA methylation is an epigenetic mechanism well known to be involved in the control of gene expression [41]. In this context we explored the association of age with gene expression in 330 NI individuals previously expression typed [18]. We observed no correlation between RNA level and age for either *DDO* or *MYOF*. Due to the location of the CpG sites identified in this study in regulatory and promoter regions we also explored associations of methylation and RNA expression for *DDO* and *MYOF*. We observed no significant associations between the methylation level of CpG sites and transcript expression in either gene. It may be that the relatively small sample size has impaired our ability to observe such correlations and as such future work will benefit from assaying a larger number of pedigree members. In addition, the Illumina HT12 array only has one probe assigned to *DDO* which presents a limitation in that a single probe does not account for other potential isoforms. Obtaining RNA-seq data in a larger sample size would allow exploration of multiple isoforms and their potential association with DNA methylation.

In this study we demonstrate the ability of the NI population to detect age-associated methylation sites. Aside from the benefit of the NI genetic isolate as a discovery cohort, there are a number of additional potential reasons for this. Firstly, the majority of studies to date have examined specific age intervals, i.e. fetal/newborn, adulthood, old age, rather than encompassing the full age spectrum. In this study we obtained a large amount of public data ranging across ~100 years of human life, and utilized both the increase in power from the number of samples as well as the increase in phenotypic trait variation (age) to our advantage. Secondly, it is important to consider the methodology used to test for association. In this study we used a machine-learning approach implemented in the R package GLMnet [17]. There have been several MWAS publications that have used the GLMnet approach to identify CpG-DNA methylation:trait associations [9,46–48]. The GLMnet package provides benefits to the identification of associations in genomic data through the implementation of an elastic-net routine. The elastic-net framework brings together two established approaches, ridge regression and LASSO, and by applying specific tuning parameters is able to overcome limitations of either method. Here we demonstrate that the GLMnet method has identified both robust DMRs and single CpGs with potential biological relevance and statistical significance which clearly validate in a separate independent cohort of public data. Further validation of our approach is demonstrated by the very good and consistent overlap of both CpG sites and genes in our analysis with those from multiple previous studies and meta-analyses.

## CONCLUSIONS

It is well established that isolated populations and large pedigrees have been beneficial for performing GWAS of complex traits. Here we have demonstrated the utility of isolated populations in the identification of methylation/epigenetic associations. Using an elastic-net framework we identified a panel of age associated DMRs in a sample of healthy NI females. This list is largely consistent with genes previously associated with aging. Interestingly our approach revealed a robust DMR in the promoter of *DDO*, a gene not previously reported in aging studies of healthy individuals. Our observations were validated in a large number of public blood samples, suggesting that this association exists in the methylomes of white blood cells, making it a potentially valuable biomarker for aging. Additionally, both *DDO* and *MYOF* are potentially relevant from a biological perspective to the mechanisms regulating aging. Further work is required to establish a role for these genes in aging, as well as additional modelling in other tissues to explore the potential of more systemic age-associated methylation profiles.

## MATERIALS AND METHODS

### Sample/cohort collection and ethics

The Norfolk Island Health Study (NIHS) was established in 2000 [16], and several collections have since occurred as part of an extended health survey [49,50]. To reduce the possibility of confounders we selected 24 individuals using stringent criteria, and investigated age at time of collection (aged 24-47 years). The NI samples in this study were restricted to females to exclude potential complications of sex in the analysis. All 24 female samples were selected as being 'healthy', meaning all selected individuals: had never smoked throughout the course of their life to date; were not on medications (i.e. hypertensive or lipid lowering), and had no adverse health events recorded in the extensive questionnaire. Additionally, as all samples belong to an extended pedigree we ensured selected individuals were as unrelated as possible, the closest relationships are second-cousins (an F 0.015625 relatedness), to reduce the influence of relatedness on association. All individuals gave written informed consent. Ethical approval was granted prior to the commencement of the study by the Griffith University Human Research Ethics Committee (ethical approval no: 1300000485) and the project was performed in accordance with the relevant guidelines, which complied with the Helsinki Declaration for human research.

### Publicly available methylation data

A total of >4700 blood samples were extracted from the Illumina 450K methylation repository, MARMAL-AID [11]. These data was filtered based on the presence of age phenotype information, reducing the final number to 2316 for which beta values for the probes of interest were obtained. These samples had an age range of 0 to 103 years of age, with a mean of 54 years of age.

### Methylation arrays and quality control

EDTA anticoagulated venous blood samples were collected from all participants enrolled in the NIHS. Genomic DNA was extracted from blood buffy coats via standard phenol-chloroform procedures. Prior to the array procedures 0.5 μg DNA from each sample was bisulfite-converted using EZ DNA methylation kits (Zymo Research Corp., USA). DNA methylation was measured at 485K CpG sites using the Illumina Human Methylation 450K BeadChip arrays. Raw intensity data (Illumina 450K idats) were loaded into R [51] using the Bioconductor minfi package [52]. Background correction and control normalisation was implemented in minfi. A further custom filter for probe quality was applied; probes were classed as failed if the intensity for both the methylated and unmethylated probes was <1,000 (based on intensities observed for negative control probes). Any probe which failed in at least one sample, was removed from the entire dataset. All probe sequences were mapped to the human genome (hg19) using BOWTIE2 [53] to identify potential hybridisation issues. 33,457 probes were identified as aligning greater than once and these were removed from the entire dataset. Additionally, we removed all previously identified cross-reactive probes [54]. Furthermore, as our sample cohort was female, Y chromosome probes were filtered from the dataset. The final number of probes after QC and filtering was 446,455. We chose to retain probes annotated to contain SNPs, with the view that SNP effects could be further explored if found to be present. All analysis was performed on beta values, calculated as the intensity of the methylated channel divided by total intensity including an offset ((methylated + unmethylated) + 100). All analyses was performed in R 3.2.3 [51], using a range of Bioconductor packages and custom scripts.

### Age association profiling

GLMnet penalised ridge-regression mixed with lasso in an elastic-net framework was used as implemented via the R package *glmnet* [17] to explore methylation association with age at time of collection in 24 health NI females. It is accepted that conventional statistical analysis procedures that test each CpG within an independent regression model suffer from multiple testing burden and reduced statistical power. To overcome this issue we choose to use the penalised regression procedures of GLMNet, which tests all markers simultaneously, i.e. in a single regression model. GLMNet was specifically designed to overcome issues of large variable number (k) and small sample size (n) and has been successfully applied to several genome-wide association studies of SNPs [55–57] and recently methylation [12]. Briefly, *glmnet* fits a generalized linear model via penalized maximum likelihood. The regularization path is computed for the lasso or elastic-net penalty at a grid of values for the regularization parameter lambda λ. The elastic-net penalty is controlled by α, and bridges the gap between lasso (α=1, the default) and ridge α=0. The tuning parameter (α=1) controls the overall strength of the penalty. The ridge penalty shrinks the coefficients of correlated predictors towards each other while the lasso tends to pick one of them and discard the others. The elastic-net penalty mixes these two; if predictors are correlated in groups, an α=0 tends to select the groups in or out together. We selected an alpha at the lower end of the range (0.05) to shift the elastic-net model more towards the penalised-regression (ridge regression), allowing us to retain more related features (CpG sites which share variance), meaning we were able to reliably detect differentially methylated regions. For the GLMnet modelling we used cross-validation to determine the optimal value of regularization parameter λ with both minimum mean squared error (MSE) and minimum MSE + 1SE of minimum MSE. The optimal λ values were then used for predictor variable selection. Equation 1 shows our implementation of the GLMnet equation in R:

age.model < −glmnet(x = t(beta.matrix), y = age, alpha = 0.05,nlambda = 425) (1)

It is important to highlight a key distinction between conventional regression modelling and the penalised regression (PR) model used in our paper: CpGs retained in the final PR model are not assigned a statistical significance (P) value as they would be in a single-marker analysis using conventional regression modelling. Instead GLMNet includes bootstrapped cross-validation for tuning and selecting the optimal lambda, as well as the selection of alpha (the penalisation parameter). Under the penalised-regression routine all predictor variables which aren't penalised to zero are retained in the overall model in the elastic-net framework. In order to 'rank' the sites we defined the absolute range for each CpG site, that is the absolute value of the largest observed beta minus the smallest observed beta for a given CpG site (Additional File: Table S1.), under the assumption that the ‘larger absolute range’ loci would have more biological relevance. Additionally we ran bayesglm (bayesian generalized linear models) from the arm package [58] for all 497 sites extracted from the model. The regression model was implemented as below:

age.bayesglm < −bayesglm(age ~CpG.site) (2)

This provided regression coefficients (signifying effect size) and p-values for each of the 497 CpG sites (Additional File: Table S1.). To test the association of the specific methylation probes in the additional public blood data the inbuilt general linear regression (*lm*) model within R was used.

### Gene expression and age in the NI cohort

We have previously reported on the collection, processing and analysis of Illumina HT-12 gene expression data for 330 NI individuals [18]. Correlation between the expression of the three available *DDO* transcripts (ILMN_1790329, ILMN_1790329, ILMN_2393461) and age was performed in R using the inbuilt pearsons correlation method (*cor*). This was also performed for the three available *MYOF* transcripts (ILMN_1810289, ILMN_3302919, ILMN_2370976).

### Overlap with previous studies

To further explore biological significance of our associated genes with aging, we downloaded the latest build of the GenAge Database of Ageing-Related Genes [20], build 18 October 11 2015. This build contained 305 human genes previously associated with age. We explored the overlap between the genes identified in our study with the 305 from this GenAge database. In addition we also compared our results to those detailed by Horvath in his 'epigenetic clock' algorithm [9], as well as a list of 1497 age associated genes recently identified in a large meta-analysis [19].

### Pathways enrichment analysis

Functional enrichment of the 422 genes representing the 497 age-associated CpG sites was performed in the ToppGene Suite webserver [21] using the ToppFun function. Bonferroni adjusted correction was used in the reporting of all pathways results (adjusted P<0.05).

### Distribution of materials and data

Due to ethics constraints restricted data access is in place to anonymised methylation and expression data. The Norfolk genetics steering committee will assess restricted data access requests via our GRC computational genetics group (interested researchers should contact grccomputationalgenomics@gmail.com).

## SUPPLEMENTARY MATERIALS TABLES AND FIGURES






