# Supplementary material for: Methylome-wide association study of whole blood DNA in the Norfolk Island isolate identifies robust loci associated with age
Source: Aging (Albany NY). 2017 Feb 28;9(3):753–66. doi: 10.18632/aging.101187 (PMC5391229; doi:10.18632/aging.101187)
Supplement: Supplementary file 1 [file aging-09-753-s001.pdf]

SUPPLEMENTARY MATERIAL

Please browse the Full text version of this manuscript to see Supplementary Tables:

Table S1. List of all GLMnet reported 497 CpG sites associated with age in the 24 healthy female Norfolk Island samples. Description of columns: IlmnID = label of Illumina 450K probe; direction = direction of effect; CHR = chromosome; MAPINFO = physical position on chromosome; gene = gene symbol; feature = genomic feature (i.e promoter, exon, etc.); Estimate = bayesglm coefficient; Std..Error = standard error; t.value = bayesglm t statistic; Pr...t.. = bayesglm p-value; range = absolute range of methylation.

Table S2. List of all significant ToppGene pathways results passing a Bonferroni threshold (adjusted p < 0.05). Description of columns: Category = GO category; ID = GO ID; Name = name of GO annotation; p-value = unadjusted p-value; q-value Bonferroni = Bonferroni adjusted; q-value FDR B&H = Benjamini and Hochberg adjusted FDR; q-value FDR B&Y = Benjamini and Yekutieli adjusted FDR; Hit Count in Query List = number of genes from study that match GO annotation; Hit Count in Genome = number of genes in specific GO pathway; Hit in Query List = specific gene symbols which match the GO annotation.

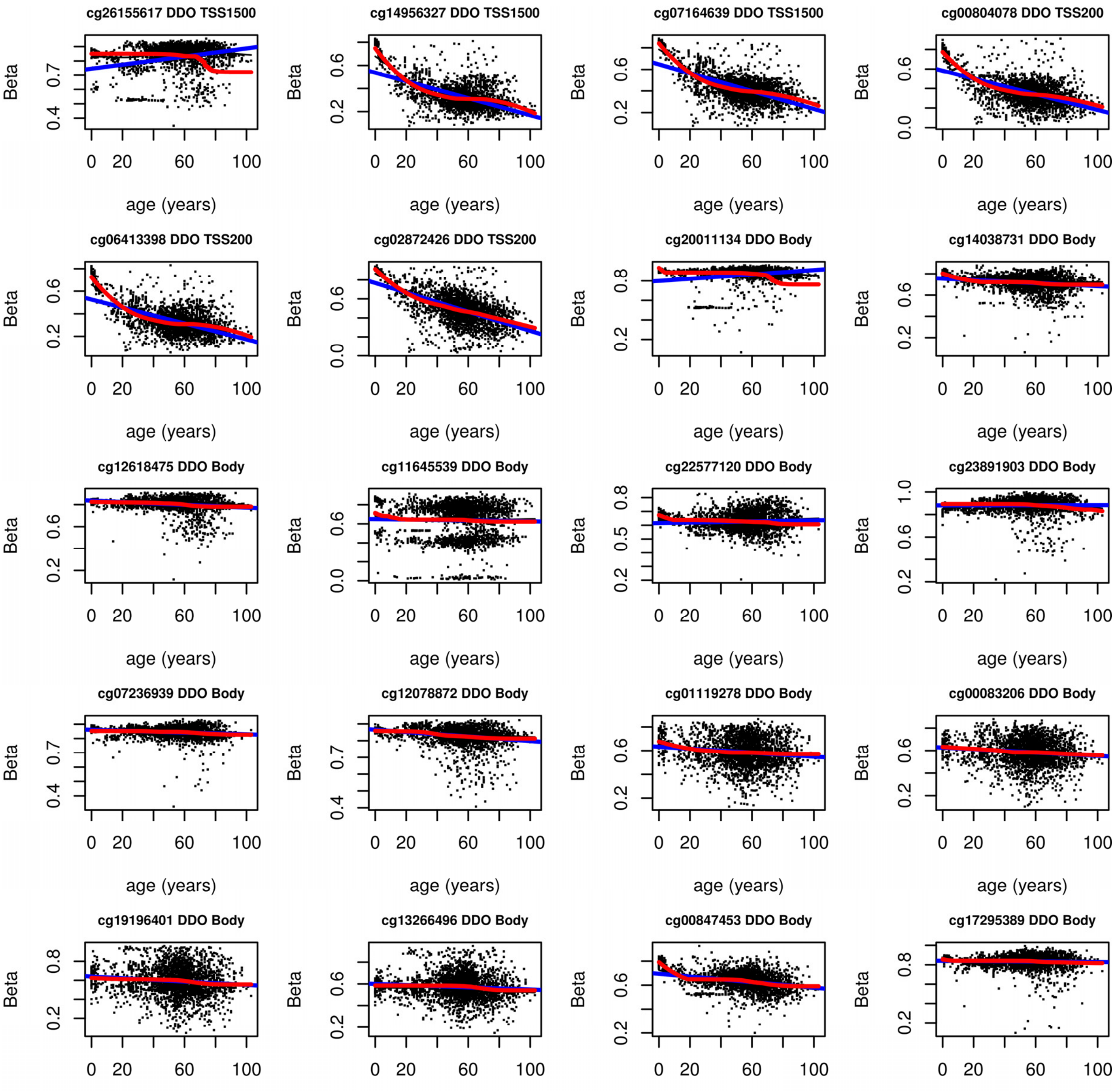

Figure S1. All *DDO* probes present on the Illumina 450K, showing CpG associations with age from the available 2316 blood samples. Probes are ordered according to genomic feature. Fitted models represented are linear regression (blue line of best fit) and loess regression (red line of best fit). The original 4 promoter CpG sites associated in the NI cohort are present.

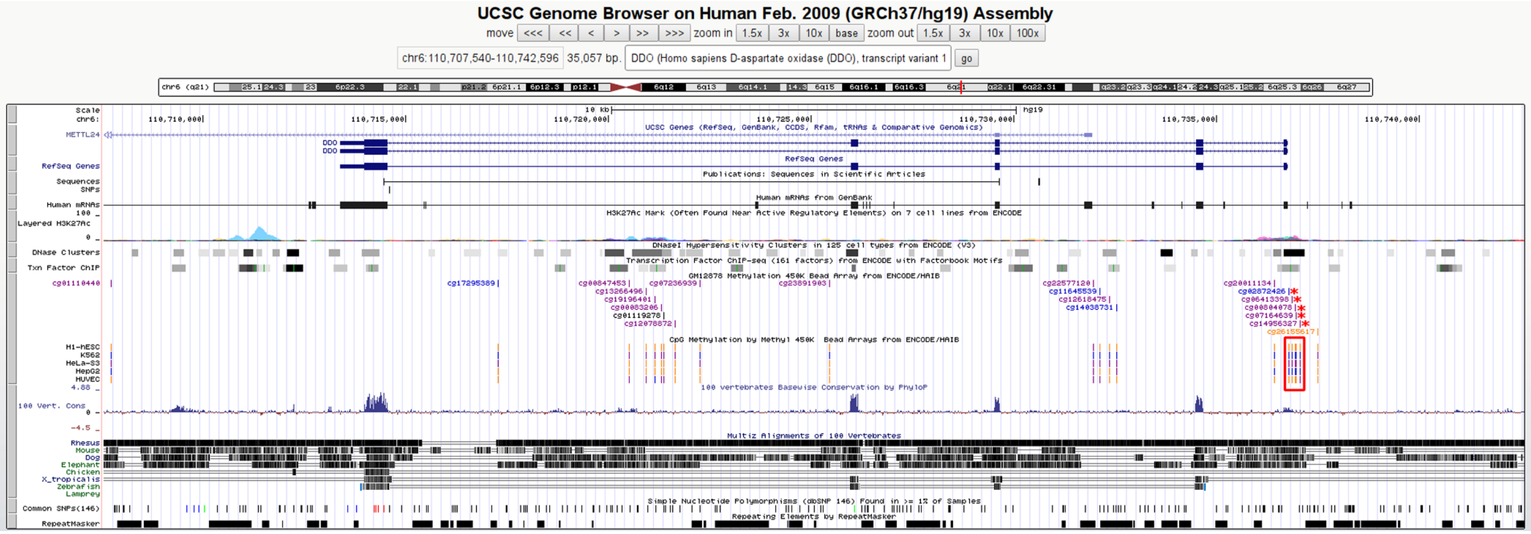

Figure S2. UCSC hg19 track snaphot of chromosome 6 centered on *DD0*. The 5 promoter associated CpG sites are indicated by a red astrix and a red box.
