# Supplementary material for: Methylome-wide association study of whole blood DNA in the Norfolk Island isolate identifies robust loci associated with age
Source: Aging (Albany NY). 2017 Feb 28;9(3):753–66. doi: 10.18632/aging.101187 (PMC5391229; doi:10.18632/aging.101187)
Supplement: Supplementary file 2 [file aging-09-753-s002.pdf]

IlmnID	direction	CHR	MAPINFO	gene	feature	Estimate	Std..Error	t.value	Pr...t..	range
cg10501210	Down	1	207997020			-44.6541838228	6.8313799314	-6.5366271927	1.41826958956076E-06	0.4388044702
cg02872426	Down	6	110736772	DDO	TSS200	-51.1488601772	9.0281608608	-5.6654794886	1.06943063758303E-05	0.383055549
cg14060519	Down	10	95222867	MYOF	Body	-55.4435123339	11.3859517299	-4.8694666594	7.23041700862993E-05	0.379698637
cg10835286	Down	2	66657913			-50.478935601	9.8607346847	-5.1191860662	3.94951156180008E-05	0.3760563109
cg11807280	Down	2	66654644			-53.3192177332	10.050078772	-5.3053532159	2.52325050935448E-05	0.3459084412
cg03274391	Down	3	22413232			-65.5541333943	11.7163989422	-5.5950752205	1.26364815324447E-05	0.338245761
cg22943590	Down	2	66648797			-54.3468946585	10.9620720622	-4.9577209811	5.83664736455066E-05	0.3297531075
cg26063719	Down	10	17273187	VIM	Body	-60.0042664225	10.9991088329	-5.4553752794	1.76212847057423E-05	0.3251561798
cg07164639	Down	6	110736958	DDO	TSS1500	-62.7191028172	12.2953101481	-5.1010590267	4.12618668933398E-05	0.3235091885
cg18646240	Up	1	40626599	RLF	TSS1500	71.956023872	14.7701820756	4.8717086562	7.19115298769276E-05	0.2913987176
cg06413398	Down	6	110736865	DDO	TSS200	-69.2625005075	13.8354232063	-5.0061714394	5.19027561389847E-05	0.2907579489
cg11693709	Down	15	40542019	PAK6	5'UTR	-73.7274497994	14.8008079471	-4.981312511	5.51233949475247E-05	0.2870471794
cg11247817	Down	5	96294344	LNPEP	Body; 5'UTR; 1stExon	-78.1512319574	16.2399473439	-4.8122835809	8.30837560028564E-05	0.2553949823
cg05523882	Down	16	70323915	AARS	TSS1500	-59.0299964639	18.0220032486	-3.2754403409	0.0034576023	0.2517026765
cg14956327	Down	6	110737053	DDO	TSS1500	-77.0692309567	14.396785564	-5.3532248997	2.24965333271087E-05	0.2437790857
cg03992323	Down	1	224023297	TP53BP2	Body; 5'UTR	-82.7607146428	16.7669668038	-4.9359383609	6.15318308052895E-05	0.2425536097
cg18079948	Down	10	20009221			-67.7716685505	15.4580482971	-4.3842319061	0.0002360094	0.2406391049
cg05412028	Down	13	95952937	ABCC4	Body	-97.1093656845	20.29289086	-4.7853884572	8.8700407247306E-05	0.2402463907
cg25413977	Down	2	66651619			-58.495864317	13.5595357927	-4.3140019844	0.0002801961	0.2371968587
cg09997082	Down	19	46170946	GIPR	TSS1500	-91.9845463605	19.0751456729	-4.8222198634	8.1100600977802E-05	0.2215917291
cg14134003	Down	9	88952877	ZCCHC6	Body	-76.9426216548	16.3503644874	-4.7058658364	0.0001076479	0.2194377657
cg09988805	Up	2	43278552			86.8928488997	15.7329554277	5.5229832245	1.49986204107768E-05	0.2179551435
cg02841912	Down	6	152955983	SYNE1	5'UTR	-79.7740591142	15.7525265938	-5.064207233	4.51041510764821E-05	0.2145259366
cg19246654	Up	7	139320460	HIPK2	Body	84.6494664327	16.6774531193	5.0756830691	4.38703364076358E-05	0.2143635966
cg12423733	Down	6	29454623	MAS1L	1stExon	-91.6203071789	18.237448463	-5.023745913	4.97412128869988E-05	0.2122497897
cg21750709	Down	3	109056897	DPPA4	TSS1500	-86.7605191066	16.8899216831	-5.1368218713	3.78497052762783E-05	0.2039111401
cg14901243	Down	19	13951845			-91.2154247219	19.0987821946	-4.7759812009	0.000090754	0.1991871975
cg05307752	Down	2	144462886	ARHGAP15	Body	-79.4718805359	17.2928207802	-4.5956574434	0.0001408312	0.1991250659
cg06289802	Down	17	72748189	SLC9A3R1	Body	-108.6244237153	18.9793271881	-5.7233021297	0.000009328	0.197773127
cg25330422	Up	17	40467382	STAT3	3'UTR	89.7816890678	22.591835722	3.9740767493	0.0006424755	0.1949500337
cg24237609	Down	12	104325856	HSP90B1	Body	-94.6061937581	20.7227767644	-4.5653241761	0.000151651	0.194625302
cg26337070	Down	2	85999873	ATOH8	Body	-83.1142784638	18.9562271509	-4.3845369546	0.0002358335	0.1942757071
cg23732483	Down	3	48965611	ARIH2	Body	-95.3378945752	20.4063419081	-4.6719737915	0.0001169157	0.1908435542
cg24919348	Up	8	100549849	MIR599; VPS13B; MIR875	TSS1500; Body	101.8186515678	19.8357630796	5.1330846794	3.819249055663E-05	0.185414433
cg05056497	Down	21	35899448	RCAN1	5'UTR; Body; TSS200	-102.5272735951	21.003172222	-4.8815137309	7.02194868219855E-05	0.185033323
cg23753748	Down	10	105212808	CALHM2	TSS1500	-107.5200966339	22.1481498064	-4.8545859394	7.49656139739213E-05	0.1824394713
cg02441474	Up	16	89165300	ACSF3	Body; 5'UTR	104.1378817855	21.2341670015	4.9042602791	6.64471923187885E-05	0.1820913855
cg03518202	Down	1	45669170	ZSWIM5	Body	-109.8202193782	23.6970563895	-4.6343401296	0.0001281505	0.1817946884
cg16971827	Up	11	119177430	CBL	3'UTR	99.6475467032	19.981255094	4.9870514257	5.43623927971475E-05	0.181683988
cg19412669	Down	1	36839270	STK40	5'UTR	-105.1426533893	19.3867518252	-5.4234280367	1.9018260058153E-05	0.1816729792
cg12476487	Up	6	145047472	UTRN	Body	101.9101581803	22.0043778421	4.6313583102	0.0001290858	0.1781770604

cg21054703	Down	13	31309032	ALOX5AP	TSS1500	-110.2850689339	21.9026051655	-5.0352489168	4.83759106724215E-05	0.1759445837
cg10453758	Down	3	132380190	UBA5; ACAD11	Body; 5'UTR; TSS1500	-98.0786933579	24.817227366	-3.9520407301	0.0006779039	0.1747195169
cg26153162	Down	2	179751100	CCDC141	TSS1500	-101.7380696435	19.4044172074	-5.2430366012	2.93066489851987E-05	0.174154916
cg16867657	Up	6	11044877	ELOVL2	TSS1500	116.0568307964	17.9252438337	6.4744910515	1.63327017069018E-06	0.1728167
cg06656994	Up	1	179713176	FAM163A	5'UTR	94.5284407006	15.8634238943	5.9588926911	5.36394187074107E-06	0.1720538342
cg03458096	Down	10	129105057	DOCK1	Body	-104.6347338579	22.5405396309	-4.6420687158	0.000125758	0.171121756
cg03725309	Down	1	109757585	SARS	Body	-105.4880985912	20.5032684737	-5.1449406092	3.71157241244495E-05	0.1709360292
cg15989436	Down	5	150465875			-92.8499268865	20.8448896869	-4.454325654	0.0001988635	0.1705100611
cg05862438	Down	7	142920096	TAS2R40	1stExon	-119.1163813045	22.9115035974	-5.1989770465	3.25841483462011E-05	0.1704508714
cg26729380	Down	6	31543655	TNF	1stExon	-128.7582774622	26.8778396987	-4.7904994935	8.76043528412763E-05	0.1678976934
cg17213713	Down	7	37617628			-99.93791025	20.1521013169	-4.9591806174	5.81603395505394E-05	0.1667849627
cg26967723	Down	5	169617918			-107.2878728685	20.4426324247	-5.2482415493	2.89421978351659E-05	0.1664621219
cg09122223	Down	11	112035175	IL18	TSS1500	-86.9422714404	19.7100183743	-4.411070035	0.0002210292	0.1664574067
cg01004762	Down	9	34956991	KIAA1045	TSS1500	-93.7577730984	21.6158898321	-4.3374468424	0.0002645941	0.1649608313
cg01951963	Up	16	85942346	IRF8	Body	114.1102949622	20.4814397465	5.5714000761	1.33673084699725E-05	0.1638265379
cg07219769	Down	22	38071455	LGALS1	TSS200	-117.5135459659	28.9555074883	-4.0584177643	0.000523063	0.1636671109
cg16351569	Down	17	43448493			-119.5635126521	28.3373157653	-4.2192956327	0.0003531539	0.1601314378
cg11988733	Up	2	176968814			144.0273571257	29.7029593226	4.8489228148	7.60042414689843E-05	0.1597504411
cg09195271	Up	1	20142497	RNF186	TSS1500	104.1603267012	21.9564283277	4.7439558541	9.8111181078074E-05	0.1589047889
cg00533891	Down	10	80919242	ZMIZ1	5'UTR	-112.5058161436	19.064827693	-5.9012238639	6.13859557961071E-06	0.1578682248
cg00227093	Down	5	149979838	SYNPO	TSS1500	-107.7104339489	25.1244516473	-4.2870760111	0.0002992527	0.1562421502
cg03976645	Up	7	16724981	BZW2	Body	125.2910136808	24.646045823	5.0836152209	4.30375220136371E-05	0.1547645836
cg12892471	Down	12	50616779	LIMA1	Body; TSS1500	-102.6007781091	19.7930075467	-5.1836881215	3.38062869684545E-05	0.1545410989
cg11879741	Down	13	104535504			-104.0451732262	27.8060073642	-3.7418235514	0.0011297084	0.1545042172
cg05901543	Up	16	89251975	CDH15	Body	132.4878034717	26.7634547083	4.9503251697	5.94223285463475E-05	0.153682365
cg25948037	Down	4	57522988	HOPX	5'UTR; Body; TSS1500	-130.7173686547	26.5204116955	-4.9289343678	0.000062586	0.1530906718
cg07747616	Down	6	7729794	BMP6	Body	-126.4024920173	27.6758837773	-4.5672432011	0.0001509424	0.152869861
cg04777348	Down	1	92952897	GFI1	TSS1500	-133.8072982561	28.3551742477	-4.7189728791	0.0001042653	0.1509533809
cg06195193	Down	13	20741008			-140.4810919478	27.7769529774	-5.0574694806	4.58448464278965E-05	0.1506980631
cg10421247	Down	12	120524653	CCDC64	Body	-131.009993387	29.0211696256	-4.514290605	0.0001717715	0.1491930724
cg05091997	Down	17	60897721			-120.6378392652	27.9810123759	-4.3114179589	0.000281971	0.1487788099
cg13719901	Down	3	46608139	LRRC2	5'UTR; TSS200	-115.2556531483	24.6689827656	-4.6720877891	0.0001168833	0.1473862941
cg09000178	Up	16	67063319	CBFB	1stExon	109.4955028198	20.0001507685	5.4747338701	1.68259486950056E-05	0.1461597982
cg03735592	Down	6	138821354	NHSL1	TSS1500; Body	-118.0981170167	22.8110645664	-5.177229527	3.43364152934352E-05	0.1459286244
cg07102001	Up	16	87734816	LOC100129637	Body	133.0150089648	23.1731919566	5.7400382828	8.96675634067975E-06	0.1449187811
cg04093047	Up	1	32827707	LOC100128071; TSSK3	1stExon; 5'UTR; TSS200	147.4041008969	21.1507059923	6.9692284007	5.38107580785355E-07	0.1440244213
cg00740914	Down	2	66652111			-102.5209806397	21.5684410225	-4.7532865511	9.59077004874226E-05	0.143940395
cg17320698	Down	11	30510489	MPPED2	Body	-114.9929893203	23.9062765783	-4.8101589113	8.35141170651538E-05	0.1439111436
cg20094837	Down	13	35515575	NBEA	TSS1500	-116.5755389764	26.5960108998	-4.3831963905	0.0002366073	0.1437203449
cg05976500	Up	17	8926895	NTN1	Body	153.7737725457	29.13056679	5.2787772258	2.68947060329969E-05	0.1435295613
cg05585551	Up	6	167314745			145.2599672598	23.657912804	6.140016174	3.51990563231677E-06	0.1434842977
cg06874172	Down	1	156901141	C1orf92	Body	-123.9476024314	25.1423807468	-4.9298275959	6.24505677434763E-05	0.1408260314

cg08310088	Down	16	28081556			-135.4541640313	26.4494037678	-5.1212558597	3.92983144362661E-05	0.1403405496
cg27252164	Up	11	2292373	ASCL2	TSS200	135.577298311	22.2638603843	6.0895682946	3.95663501668452E-06	0.1393960343
cg06545367	Up	7	110731527	LRRN3; IMMP2L	5'UTR; 1stExon; Body	143.294863474	29.4922457705	4.8587301418	7.42146404060797E-05	0.1388159916
cg15335436	Up	5	54516372			152.0954808521	28.5059947186	5.3355612514	2.34691677715141E-05	0.1360622011
cg02867102	Down	17	62398693			-143.1749644205	34.1766185562	-4.1892665357	0.0003800344	0.1356345962
cg18506678	Up	6	85482785			134.7195596496	35.9957998185	3.7426466512	0.0011274581	0.1356226614
cg09695307	Up	10	63993920	RTKN2	Body	128.75354355	25.2454247371	5.1000743656	4.13600955904787E-05	0.135254096
cg08415592	Down	22	36648973	APOL1	TSS200	-141.9650585136	32.4140658681	-4.379736226	0.0002386163	0.1335031455
cg19518666	Down	4	157692845	PDGFC	Body	-129.4295881807	26.5871922756	-4.8681179584	7.25414087352795E-05	0.1334814485
cg14714046	Up	6	41341654			134.8806031029	20.9134607638	6.4494635597	0.000001729	0.1328322962
ch.2.105901354F	Down	2	106534922			-145.6820657395	30.3527433271	-4.7996342265	8.56793007805806E-05	0.1325974895
cg08014499	Up	11	2162545	IGF2AS; INS-IGF2; IGF2	Body; 5'UTR; TSS1500	162.6517729923	31.3216986093	5.1929422801	3.30611074037143E-05	0.1317790236
cg02107844	Down	15	92612836	SLCO3A1	Body	-138.0656344749	29.8039815132	-4.63245605	0.0001287407	0.1317367046
cg01765174	Down	9	100880960	TRIM14	Body	-128.8603654567	31.9715764358	-4.0304664274	0.0005599669	0.1311237855
cg07553761	Up	3	160167977	TRIM59	TSS1500	139.3867680071	32.2678369991	4.3196811739	0.0002763343	0.129648565
cg25838150	Down	11	77259596			-171.5522708674	38.4681118836	-4.4595968574	0.0001963193	0.1294668966
cg10535353	Up	2	200323432	SATB2	5'UTR	148.8366112792	25.256296367	5.8930497614	6.25728166922785E-06	0.1294211001
cg23431721	Down	3	50387780	TUSC4; CYB561D2	Body; TSS1500	-149.0678739969	35.4033727232	-4.2105557333	0.0003607754	0.1278429392
cg15198193	Up	1	180914866	KIAA1614	3'UTR	145.2393096724	30.6644036456	4.7364139656	9.99294344626092E-05	0.1272459054
cg19457770	Up	2	113742967	IL1F9	3'UTR	107.5415408126	24.3623809319	4.4142459275	0.0002193207	0.1265013781
cg21572722	Up	6	11044894	ELOVL2	TSS1500	165.3940901989	29.9937983009	5.5142762694	1.5312791699356E-05	0.1261101443
cg09840122	Down	12	98926723	TMPO	Body	-128.0613110529	30.1486165942	-4.2476679039	0.0003295023	0.1252580088
cg18431970	Down	19	2001580	BTBD2	Body	-136.8764659402	33.48712391	-4.0874357054	0.0004873089	0.125008206
cg19120717	Up	6	41343647			148.7357854919	32.9876220032	4.5088362379	0.0001740746	0.1249723003
cg08810238	Down	17	27225046	FLOT2; DHRS13	TSS1500; 3'UTR	-170.7193102697	31.3821602996	-5.4400114154	1.8279608159264E-05	0.1249659654
cg08336641	Up	18	6414602	L3MBTL4	5'UTR	153.1115940067	26.3743949928	5.8053120858	7.68863892821321E-06	0.1232532118
cg21635307	Down	6	90319877	ANKRD6	Body	-145.6217149199	33.1035582563	-4.39897469	0.0002276588	0.1231270297
cg00182994	Down	16	2234571	CASKIN1	Body	-140.4545293838	27.0442504922	-5.1935079297	3.30161023935432E-05	0.122830379
cg11637721	Down	11	65486928	KAT5; RNASEH2C	3'UTR	-155.4152270556	25.3683286964	-6.1263486813	3.63312196938255E-06	0.121793456
cg09230679	Up	12	116586898	MIR620; MED13L	TSS1500; Body	162.0286201556	29.393863478	5.5123281183	0.000015384	0.1212270885
cg12912022	Up	7	62554288			152.3944204067	34.7699091763	4.382939847	0.0002367557	0.1208162407
cg15745401	Down	11	72974126	P2RY6	TSS1500	-166.862524849	36.6758739173	-4.5496536831	0.0001575638	0.1206619268
cg06458258	Up	16	1440604			138.6145526125	31.1081610727	4.4558902819	0.0001981048	0.120076202
cg10286673	Down	X	107069680	MID2	Body	-180.5911219537	30.4934195612	-5.9222981401	5.84307778216477E-06	0.1198830538
cg09809672	Down	1	236557682	EDARADD	TSS1500; 5'UTR; 1stExon	-151.0607432867	25.9479884783	-5.8216745168	7.39840699709603E-06	0.1192295375
cg18500714	Up	14	100706288	YY1	Body	131.5984008492	23.0293598709	5.7143751102	9.52675821133643E-06	0.119157451
cg16232504	Up	1	99127707	SNX7	Body; 5'UTR	160.1497906891	25.4209006613	6.2999259083	2.4342972216394E-06	0.1178503767
cg02609880	Down	1	156611087	BCAN	TSS1500	-186.5308927468	39.1251393029	-4.7675457793	9.26362102275936E-05	0.1177693063
cg25994988	Down	11	122652382	UBASH3B	Body	-165.522054694	32.2504915351	-5.1323885874	3.82566842858126E-05	0.1177648931
cg26417554	Down	3	9886232	RPUSD3	TSS1500	-115.3553546471	28.0093698301	-4.1184559077	0.0004517742	0.1176387559
cg21280392	Down	17	47304116	PHOSPHO1	5'UTR	-179.6080174178	33.0702929906	-5.4310984626	1.86729112643411E-05	0.1175200508
cg00636124	Up	16	27215281	JMJD5	5'UTR; TSS200	140.6670516234	25.3702215457	5.5445732458	1.4247552293513E-05	0.1162555345

cg04748546	Down	19	49696260	TRPM4	Body	-170.3378288022	34.6703183886	-4.9130736814	6.5041147025033E-05	0.1161830927
cg14445047	Down	9	139332115	INPP5E	Body	-161.5887843693	32.8964374455	-4.9120451002	6.52036815570352E-05	0.1161703303
cg16899036	Down	19	19052705	HOMER3	TSS1500	-173.7870141507	38.5821339145	-4.5043390948	0.0001759968	0.116128926
cg07781401	Up	17	19773621			146.4701381011	29.7846169181	4.9176438463	6.43239086451E-05	0.1154191739
cg17193921	Up	22	50683583	TUBGCP6	TSS200	170.7673159276	30.6279387372	5.5755406001	1.3236461079414E-05	0.1152776575
cg08616943	Down	7	130552600			-161.7227843958	41.1417060762	-3.930872096	0.0007137612	0.1147959478
cg02257517	Down	9	136340262	SLC2A6	Body	-195.4031008434	30.2413377992	-6.4614569018	1.6824531353027E-06	0.1143408701
cg07855319	Down	16	69123283			-126.8855605947	27.115286808	-4.6794843622	0.000114795	0.113681582
cg21579209	Down	7	138620362	KIAA1549	Body	-152.779581241	31.5787830551	-4.8380452462	7.80400946813795E-05	0.113397153
cg17523868	Up	17	27313033	SEZ6	Body	195.9656347093	38.9891030585	5.0261642186	0.000049451	0.1129989717
cg21467614	Down	6	31543638	TNF	1stExon	-169.931802303	35.2504214375	-4.8207027143	8.14002892636117E-05	0.1129434125
cg07667295	Up	14	64320118	SYNE2	5'UTR	144.3269331053	25.4549791746	5.6698900484	1.05832600890561E-05	0.1128725686
cg23919111	Down	14	95983256			-162.662042002	33.7321404476	-4.8221678151	8.11108638183954E-05	0.1124153503
cg05058354	Down	7	1513548	INTS1	Body	-196.8353290662	38.0139102404	-5.1779816341	3.42742509282385E-05	0.1123424002
cg19761273	Down	17	80232096	CSNK1D	TSS1500	-197.4961795622	29.5742234661	-6.6779836092	1.03059482261951E-06	0.1118206854
cg10257332	Down	3	47869137	DHX30	Body	-179.9290802879	36.7820130734	-4.8917681566	6.84928476056855E-05	0.1105574401
cg10137837	Up	17	6926742	BCL6B	5'UTR	147.1143301193	27.0811726844	5.4323471082	1.86172986442499E-05	0.1104330724
cg04084597	Up	11	75479652	DGAT2	TSS200	156.4618480295	25.454592617	6.1467040696	3.46582540924233E-06	0.1096614875
cg00958578	Up	16	103715	SNRNP25; POLR3K	TSS200	170.7996020524	29.7285464172	5.7453062002	0.000008856	0.1095296517
cg27130665	Down	11	40316514	LRRC4C	TSS1500	-152.6269837464	36.0410054785	-4.2348148094	0.0003400143	0.1089368886
cg27326306	Up	16	66442374			169.2732335047	36.2219372788	4.6732241901	0.00011656	0.1083701659
cg01042948	Down	2	101319752			-163.562651909	36.2197944248	-4.5158360092	0.0001711246	0.1068754907
cg18934443	Down	7	149569776	ATP6V0E2; LOC401431	TSS1500; Body	-176.838155365	29.5085012125	-5.9927867597	4.95593592570754E-06	0.106837952
cg07599881	Up	1	5907508			136.443893763	31.2759649498	4.3625798271	0.0002488325	0.1064506812
cg05247809	Up	17	66194706	LOC440461	TSS200	193.8135265099	35.8038266069	5.4132070473	1.94885464163592E-05	0.1063193211
cg03296370	Down	3	39323216	CX3CR1	1stExon; TSS1500; 5'UTR	-176.6100373666	36.2771614924	-4.8683532587	0.0000725	0.1061055661
cg21756806	Down	11	46638289	KIAA0652; HARBI1	TSS1500; 5'UTR	-194.295021037	44.4645828898	-4.3696580157	0.0002445656	0.105724419
cg08301518	Down	19	3672411	PIP5K1C	Body	-184.7767582646	33.7626813921	-5.4728105306	1.69033091280192E-05	0.1056383557
cg19240052	Up	22	42475693	C22orf32	TSS200	221.9733878555	39.3392649254	5.6425403036	1.12912724413211E-05	0.1054669999
cg26064535	Up	1	29138043	OPRD1	TSS1500	165.6728927446	36.3630374977	4.5560795837	0.0001551117	0.1041520958
cg19414630	Down	8	48648874			-145.6542730626	30.6605566368	-4.7505423593	9.65505281358296E-05	0.104066306
cg04328762	Up	22	19706186	SEPT5	Body	191.9660689003	32.1914112796	5.963269744	5.30938164014294E-06	0.1038279166
cg19942298	Down	20	2516502	TMC2	TSS1500	-196.6948703051	47.0661267659	-4.179117421	0.0003895732	0.1036417622
cg01619796	Up	21	38790095	DYRK1A	5'UTR; TSS1500	189.0090555684	42.8951311782	4.4063055731	0.0002236172	0.1025695906
cg06218726	Up	14	104583740	MIR203	TSS200	157.8451320786	27.9000983883	5.6575116647	1.08979305815239E-05	0.1019525524
cg04553410	Up	7	150864885	GBX1	TSS200	176.2421800418	27.301371552	6.4554331897	1.70569276766702E-06	0.1017113234
cg16637224	Down	2	130538947			-162.0891624142	41.5224868703	-3.9036477492	0.0007626533	0.101554243
cg14001161	Down	X	24713101	POLA1	Body	-167.0229555403	37.533529008	-4.4499667352	0.0002009923	0.1012882687
cg17737621	Up	20	21372480			190.4181259428	36.7162343141	5.186210664	3.36014917658588E-05	0.1011107681
cg10087036	Down	16	19130461	ITPRIPL2	1stExon; Body; 3'UTR	-163.8581899385	41.5545950659	-3.9432026634	0.000692652	0.100406056
cg10278149	Down	2	208016923	KLF7	Body	-170.4547440162	36.5854533694	-4.6590851915	0.0001206473	0.1004039892
cg01510278	Down	16	11456238			-197.718037118	44.1441648776	-4.4789166964	0.0001872701	0.1003870959

cg14210872	Up	1	155294935	RUSC1; C1orf104	Body; TSS1500	235.7303469579	47.0161481541	5.0138166611	0.000050951	0.1002382202
cg24211504	Up	6	100903575	SIM1	Body	213.0051499523	30.4011321497	7.0064874197	4.9557499077634E-07	0.09999376
cg08291854	Down	5	608798			-208.629654259	38.7401158016	-5.3853647554	2.08304079332246E-05	0.0999442103
cg23962478	Up	22	50354086	PIM3	TSS200	184.5974922449	25.6281114085	7.2029299897	0.000000322	0.0991367748
cg07021644	Up	22	45680879	UPK3A	1stExon; 5'UTR	185.9044477097	37.5364738017	4.9526348344	5.90905366009423E-05	0.0989566074
cg13390975	Up	5	34915890	BRIX1; RAD1	1stExon; TSS200	172.6123486738	25.1608518557	6.8603539206	6.85192846953332E-07	0.0982942299
cg15868105	Up	22	30116953	CABP7	Body	190.3953247906	33.9611691463	5.6062653194	1.23053274255489E-05	0.0981076878
cg08062273	Up	3	45641120	LIMD1	Body	206.9149826761	38.7377951232	5.3414238476	2.31417068666221E-05	0.0978402841
cg22265644	Up	20	21494716	NKX2-2	TSS200	226.7688746603	38.3226274558	5.917362397	0.000005911	0.0974434642
cg17288256	Down	12	129300720	MGC16384; SLC15A4	TSS1500; Body	-150.3277344522	36.3610023835	-4.1343121641	0.0004346192	0.0973496971
cg08220136	Up	13	79168325			223.6605441008	46.9484901902	4.7639560547	9.34490598217473E-05	0.0972906894
cg16290275	Down	1	208042910			-178.9589131198	39.7997836137	-4.4964795502	0.0001794074	0.0972427087
cg16762684	Down	18	74820493	MBP	5'UTR	-161.892917586	38.1317087164	-4.2456245218	0.0003311515	0.0969877198
cg14208102	Down	3	48507165	TREX1	TSS200	-157.0940602265	35.3901993367	-4.4389142523	0.0002064931	0.0968865447
cg01812894	Down	9	75568506	ALDH1A1	TSS1500	-160.8998786792	39.1519337883	-4.1096278807	0.0004616158	0.0966923929
cg14225031	Up	9	129387848	LMX1B	Body	166.6342787551	35.4821443939	4.6962854585	0.0001101901	0.0966730267
cg25616514	Down	3	11798996			-180.6797759891	42.6595420903	-4.2353894846	0.0003395372	0.0965654722
cg24436906	Up	2	242498081	BOK	TSS200	180.2159044626	35.4250215249	5.0872489756	4.26613647383768E-05	0.0964960503
cg17327171	Down	2	161156221	RBMS1	Body	-197.2886534362	43.8939438682	-4.4946668276	0.0001802033	0.0963606005
cg25090514	Up	5	2038743			178.3327858714	36.9580216344	4.8252795465	8.04995892560679E-05	0.096125639
cg02369193	Down	19	11587407	ELAVL3	Body	-245.0366324796	48.6600468027	-5.0356842745	0.000048325	0.0956443228
cg23091758	Up	11	9025767	NRIP3	TSS200	226.6029197527	40.5094674253	5.5938261882	0.000012674	0.0948254106
cg16341592	Up	3	112931182	BOC	TSS200	204.646955446	31.4268406636	6.5118526433	1.5002882172704E-06	0.0942281735
cg04704634	Down	5	131396204	IL3	TSS200	-182.4152570517	41.352445696	-4.4112326123	0.0002209414	0.094226455
cg03179542	Down	7	2647333	IQCE	Body	-158.8806918721	39.7999907464	-3.9919781109	0.0006150524	0.0940621408
cg24818699	Down	13	43355514	C13orf30	TSS200	-164.9950886119	42.1668957591	-3.9129057438	0.0007456654	0.093972953
cg07490485	Down	14	50432614			-186.8314626761	42.7459326839	-4.370742453	0.0002439184	0.0939570427
cg07057177	Up	7	132261393	PLXNA4	TSS200; 5'UTR	212.50363138	32.7450162076	6.4896480745	1.57792361886336E-06	0.0929490455
cg06365623	Down	2	55389666			-203.3300476232	46.6960755535	-4.354328393	0.0002539008	0.0909988013
cg15774153	Up	11	69518773	FGF19	1stExon; 5'UTR	235.974254567	48.3510975205	4.8804322274	7.04041316979585E-05	0.0909133598
cg20841134	Down	2	64247030	VPS54	TSS1500	-206.7074803118	40.6511195841	-5.0849148173	4.29026071587642E-05	0.0902603858
cg22591103	Up	3	159944037	LOC401097	1stExon	231.1886074448	36.2620428644	6.3754987084	2.04712377202302E-06	0.089719106
cg23758016	Up	17	73521635	LLGL2	TSS200	240.6932688544	37.7643463326	6.3735584547	2.05623046304834E-06	0.0893475952
cg27486786	Down	7	99074036	ZNF789	5'UTR	-232.7879679103	44.428807016	-5.2395727805	2.95517603533689E-05	0.0890824642
cg00407659	Down	5	150538414	ANXA6	TSS1500	-228.9776196609	38.6202643261	-5.9289500902	5.7528444499479E-06	0.0887832065
cg06110297	Up	7	22589830			222.6596446613	54.5982624517	4.0781452497	0.0004984831	0.0885855642
cg00730820	Down	1	27694390	MAP3K6	TSS1500	-212.928513986	36.0860525389	-5.900576511	6.14791072569663E-06	0.088234245
cg24401219	Up	6	72892597	RIMS1	Body	245.4153818515	47.9428250817	5.1189178242	3.95206936511118E-05	0.0882214598
cg00841151	Down	7	100612136	MUC12	TSS1500	-202.0222995243	44.7588483286	-4.5135723341	0.0001720731	0.08821914
cg24751707	Down	14	101830869			-230.7988141319	54.7447566775	-4.2159072054	0.0003560895	0.0880250085
cg08481246	Up	11	8284550	LMO1	Body	220.9461569513	38.1053749191	5.7982937426	7.81665901423157E-06	0.0874727475
cg14317285	Up	3	181444870	SOX2OT	Body	221.4944655615	46.2692131713	4.7870808769	8.83359484859925E-05	0.0870904844

cg10548254	Down	22	50656772	TUBGCP6	Body	-245.9321409905	52.5587246178	-4.6791877615	0.0001148781	0.0868877646
cg27378767	Up	10	60027795	IPMK; CISD1	TSS200; TSS1500	268.1398867625	48.1963804359	5.5634859783	1.36210784373352E-05	0.0868199374
cg27213509	Up	2	176947228	EVX2	Body	251.7960383571	47.0504875207	5.351613801	2.25835263983565E-05	0.0867020606
cg00917569	Up	16	51187388			230.0473082227	49.1502999465	4.6804863546	0.0001145151	0.0866732651
cg01373189	Down	13	33002820	N4BP2L1	TSS1500	-199.5071341958	37.485151725	-5.3222976303	2.42274767664518E-05	0.0864695185
cg02835038	Up	21	47743854	PCNT; C21orf58	TSS200	198.6257966419	31.6955040983	6.2666867839	2.62758090550791E-06	0.0862242985
cg12582330	Down	1	2473224			-202.8678187448	33.646267933	-6.029430044	4.55033058488434E-06	0.0862196004
ch.2.207814544R	Down	2	208106299			-188.121079585	39.1307238543	-4.8075031856	8.40552068961859E-05	0.0860938368
cg10857887	Up	X	148622612	LOC100131434; CXorf40A	TSS1500; 5'UTR; 1stExon	253.2132885565	38.5440388897	6.5694539506	1.31661397264246E-06	0.0860260997
cg08096946	Up	3	122514224	DIRC2	1stExon	216.6827369439	35.9816741301	6.0220304414	4.62941406826076E-06	0.0853763475
cg05766107	Down	11	63702235			-203.5938809659	45.497322571	-4.4748541114	0.0001891376	0.0852186716
cg11127482	Up	8	145551156	DGAT1	TSS1500	263.3931208952	47.524550645	5.5422537893	1.43263735832959E-05	0.0852052047
ch.1.839062R	Down	1	25282539	RUNX3	Body	-193.3049489626	45.3530572756	-4.2622253179	0.0003179878	0.0849610373
cg21296230	Up	15	33010536	GREM1	5'UTR	226.8475792594	44.3873892871	5.1106312604	4.03191654806801E-05	0.084793434
cg15574642	Down	17	48474021	LRRC59	Body	-193.5260976883	44.5266321957	-4.3462999141	0.0002589313	0.0847299485
cg26473844	Up	5	37834909	GDNF	5'UTR; TSS200	222.3465595981	34.4215895786	6.4595087653	1.6899334159334E-06	0.0845095178
cg04678392	Up	15	68114548			225.2086099927	47.1323095454	4.7782213977	9.02606871261993E-05	0.0844999856
cg14334310	Down	14	103558502			-220.5499297667	52.4474151576	-4.2051630019	0.0003655598	0.0839362153
cg06205331	Down	3	124303268	KALRN	Body; TSS1500	-251.5211894314	55.0366783854	-4.5700648515	0.0001499065	0.0835847107
cg16046667	Down	20	35422698	C20orf117	Body	-211.8322902491	42.7022046901	-4.9606874349	5.79483136302808E-05	0.0834710742
cg04533881	Up	14	50583218	C14orf138	1stExon	195.8131149512	34.1607793503	5.732103268	9.13623371481032E-06	0.0833265599
cg08483876	Up	8	145910754			221.6275827735	31.9805263657	6.9300792688	5.86850812706036E-07	0.0826996727
cg15589354	Down	1	229760606	URB2; TAF5L	TSS1500; 5'UTR	-193.4036040793	46.0596767289	-4.1989787557	0.0003711243	0.0824401404
cg06682330	Down	17	80200634			-207.2733725755	39.5299656857	-5.2434493423	2.92775797719101E-05	0.0822439811
cg25427880	Up	10	102322128			263.627979932	37.7148876227	6.9900242729	5.1392569900759E-07	0.0821792225
cg02461956	Up	11	134093940	NCAPD3; VPS26B	1stExon; TSS1500; 5'UTR	187.1062638654	33.8918730101	5.5206823125	0.000015081	0.081879374
cg14361627	Up	7	130419116	KLF14	TSS1500	211.7398252807	41.0739183836	5.1550919321	3.62182176896864E-05	0.0818046014
cg12179179	Up	21	28217735	ADAMTS1	TSS200	280.3818094791	42.1807419073	6.6471521552	1.1046897746354E-06	0.0815155068
cg25311470	Down	7	107950866	NRCAM	5'UTR	-196.2667045732	45.3390204379	-4.3288695406	0.0002701987	0.0808008404
cg11647481	Up	7	4781704	FOXK1	Body	220.2766893714	46.8647858628	4.7002602341	0.0001091281	0.0803630942
cg14868996	Up	X	68758302			249.0489130065	44.3712615113	5.6128427393	1.21148113143034E-05	0.0800201477
cg18406197	Up	3	13590450	FBLN2	TSS200	261.5336009414	51.1486616278	5.1132051674	4.00694178563387E-05	0.079676612
cg11368617	Down	11	133582402			-262.656450699	50.43676556	-5.2076386696	3.19117276545599E-05	0.0791089192
cg09458197	Down	4	2536968			-202.9652551874	43.6882531938	-4.6457626559	0.0001246304	0.0790272695
cg07240689	Down	10	105205887	PDCD11	3'UTR	-205.4323315691	42.6686576842	-4.8145956006	0.000082618	0.0786705687
cg25318296	Up	13	100547785	CLYBL	3'UTR	270.2700827456	47.8933972059	5.6431595692	1.12747195628289E-05	0.0782785995
cg08659183	Down	3	141032945			-256.7772325891	48.1293238385	-5.3351514651	2.34922317914386E-05	0.0778264995
cg26126740	Up	20	42574332	TOX2	5'UTR; TSS1500; Body	193.4738829023	38.6731625899	5.0027944431	5.23288549140226E-05	0.0778070138
cg05244952	Up	10	108927230			236.474649026	48.2214772306	4.9039279302	6.65008097278481E-05	0.0777205188
cg01547051	Up	7	150777654	FASTK	Body	260.6284850972	43.5252014659	5.987990321	5.01169305899123E-06	0.077114319
cg11214555	Up	19	36337033	NPHS1	Body	286.5859055147	53.7748009199	5.3293717617	0.00002382	0.0767660677
cg13569583	Down	6	150183531	LRP11	Body	-245.8383617725	57.2871503231	-4.2913351491	0.0002961543	0.0762192789

cg14428048	Up	7	121940344			297.0194323372	52.8821945892	5.6166245491	1.2006631799386E-05	0.0760763689
cg06521331	Down	12	34319734			-324.253642889	63.4254806143	-5.1123561028	4.01516295735204E-05	0.0755214864
cg18064714	Up	7	20824556	SP8	Body	227.3265409345	45.7835155675	4.9652486952	5.73112400524519E-05	0.0754954424
cg12991050	Up	5	87979504	LOC645323	Body	219.9763099804	42.6903979566	5.152828751	3.64163897258024E-05	0.0752860572
cg12426141	Up	15	57210872	TCF12; LOC145783	5'UTR; TSS200; 1stExon	241.6839267299	38.9782541627	6.2004810611	3.06068623380898E-06	0.0748388456
cg16664394	Up	5	54527671	CCNO	Body	318.6488749289	48.4648642682	6.5748430278	1.30065285865543E-06	0.0747875835
cg18120578	Up	12	77459407	E2F7	TSS200	241.1253206469	47.5525104802	5.0707169445	0.0000444	0.0745173617
cg09233651	Up	12	114838567	TBX5	Body	207.2307118735	46.1721313799	4.4882206145	0.0001830627	0.0735947046
cg12411312	Down	3	50608551	HEMK1	Body	-315.2470071439	62.6137724595	-5.0347869927	0.00004843	0.0734267419
cg02964724	Up	11	2292361	ASCL2	TSS200	237.5064213822	40.2536547763	5.900244902	6.15268802627806E-06	0.0732305315
cg01020263	Down	16	50732073	NOD2	Body	-193.8816366957	45.0759886633	-4.3012176204	0.0002890879	0.0731972565
cg17029062	Up	3	32859445	TRIM71	TSS200	278.6527754831	45.9797609966	6.0603354485	4.23465358078056E-06	0.0731943243
cg17721618	Down	15	42376692	PLA2G4D	Body	-250.8329794148	54.0370355384	-4.6418715778	0.0001258184	0.0729913411
cg27535757	Up	6	30434129			268.7134918406	41.9427087493	6.4066794886	1.90630578724715E-06	0.0727422503
cg11070420	Down	11	133804743	IGSF9B	Body	-236.1603183689	54.5259631585	-4.3311535402	0.0002686948	0.0718312353
cg04866628	Up	4	187026225			280.486287038	53.239967937	5.2683406453	2.75774450345296E-05	0.0716929861
cg16797421	Down	6	147428486			-299.0747231598	53.9365570058	-5.5449353789	1.42352858968448E-05	0.0715612216
cg02583938	Up	22	19748777	TBX1	Body	294.5612629731	56.3293113655	5.2292715077	3.02930591394049E-05	0.0715332121
cg08502703	Up	21	45138838	PDXK	TSS200	279.7817030884	55.9850153715	4.9974390689	5.30118395519502E-05	0.071490181
cg26070379	Down	1	9667405	TMEM201	Body	-328.2083344137	52.9140647473	-6.2026672111	3.04527974275376E-06	0.0707998138
cg01782371	Up	13	25946347	ATP8A2	1stExon; 5'UTR	250.8387925274	50.2963451852	4.9872170951	5.43405827252774E-05	0.0703698654
cg02900034	Down	8	145535640	HSF1	Body	-224.3095229744	59.3014071561	-3.7825328897	0.0010235552	0.0703483915
cg02749735	Up	3	185912253	DGKG	Body	292.7101489587	58.4370075757	5.0089859338	5.1550311982172E-05	0.0700257372
cg13557178	Up	13	22178108	EFHA1	1stExon	273.1418542995	43.8297708489	6.231879588	2.84685405910435E-06	0.0700140747
cg02547025	Up	2	30454275	LBH	TSS200	238.0681426285	43.9129657653	5.4213633372	1.91123228996573E-05	0.0700091915
cg20248822	Down	1	19922867	C1orf151	TSS1500	-221.511701919	54.1027601535	-4.0942772844	0.0004792398	0.0698567187
cg14957718	Up	3	50243260	SLC38A3	5'UTR	210.586470383	36.1517712883	5.8250664595	7.33965249691706E-06	0.0696667904
ch.2.30415474F	Down	2	30561970			-235.1709423138	50.099958209	-4.6940347002	0.000110796	0.069396164
cg20338600	Up	5	98110229	RGMB	Body	310.6766404521	52.5165725437	5.9157828739	5.93285659487777E-06	0.0693055158
cg17859448	Up	1	101703749	S1PR1	5'UTR	264.7002916931	47.4401916777	5.5796631997	1.3107473911349E-05	0.0690777521
cg10872057	Down	10	465460	DIP2C	Body	-287.5562144889	52.8446564447	-5.441538158	1.8213086141413E-05	0.0689936749
cg08202743	Up	11	34182570	ABTB2	Body	249.2143067139	57.6849038709	4.3202690824	0.0002759376	0.0689140755
cg14405813	Up	7	139414573	HIPK2	Body	246.2088385237	51.3885492435	4.7911225778	8.7471669923872E-05	0.0680829096
cg00894289	Up	11	33890917	LMO2	Body; 1stExon	272.3050405504	55.2919073624	4.9248624897	6.32072477672216E-05	0.0678686987
cg00518190	Down	1	6294768	ICMT	Body	-273.3815884147	60.4850947787	-4.5198174759	0.0001694691	0.0677329687
cg22320000	Up	1	245027629	HNRNPU	5'UTR; 1stExon	282.6668380996	41.669812787	6.783491914	8.13394796796907E-07	0.0675262836
cg21176755	Down	9	99418634	C9orf21	TSS1500	-209.7882828448	54.3114134278	-3.8626923809	0.0008425221	0.0674363361
cg00471989	Down	16	30380183	TBC1D10B	Body	-184.9906796771	59.8885818994	-3.0889140101	0.0053622361	0.0673379273
cg08644498	Down	1	46502608			-296.0337661584	56.3263157032	-5.2556919881	2.84284943897508E-05	0.0672115479
cg01725318	Up	4	141348737	CLGN	1stExon; 5'UTR	291.272951762	55.9664752573	5.2044183669	3.2160069608098E-05	0.0670615133
cg23929317	Down	1	201123992	TMEM9	TSS1500	-260.8703462801	46.7261896716	-5.5829578254	1.30053116346788E-05	0.0670349797
cg00957665	Down	10	104406345	TRIM8	Body	-241.4562975144	57.5969798877	-4.1921694156	0.0003773491	0.0666860567

cg04275707	Up	20	20033092	CRNKL1; C20orf26	Body; TSS200	274.9690864867	48.069768508	5.7202082519	9.39642122405915E-06	0.0665270809
cg06020238	Up	10	35928558	FZD8	1stExon	277.9003939158	54.270443949	5.1206582017	0.000039355	0.0664334787
cg11863248	Down	7	132937434	EXOC4	TSS1500	-324.4969165663	60.0829433833	-5.4008159104	2.00745526606206E-05	0.0663456154
cg20754324	Down	22	38381626	SOX10	TSS1500	-280.6088565032	64.3244623323	-4.3623972332	0.0002489436	0.0661102194
cg01082498	Up	11	68608225	CPT1A	5'UTR	254.0077833023	41.5249082381	6.116998064	3.71271594748764E-06	0.0660682792
cg24756528	Up	16	4466888	CORO7	TSS1500	281.300869509	49.2681359264	5.70959027	0.000009635	0.0659005428
cg01797043	Down	16	2004686	RPL3L	TSS200	-268.1523735943	62.2276937204	-4.3092127888	0.0002834946	0.065866995
cg02151752	Down	6	32798417	TAP2	Body	-331.1647983255	62.5308403147	-5.2960234767	2.58038284317709E-05	0.0655368834
cg09111258	Up	17	46114933	COPZ2; MIR152	Body; TSS1500	248.0639182036	40.8587465494	6.0712561973	4.12852975645259E-06	0.0653792449
cg03671660	Up	21	40554907	PSMG1	Body	250.4957187481	40.2795181439	6.2189353371	2.93309447135693E-06	0.0649381112
cg10536999	Down	7	26193109	NFE2L3	Body	-264.5994138526	66.7167468495	-3.9660119288	0.0006552233	0.0645080159
cg22538338	Up	13	42748498	DGKH	Body	270.2151490503	57.7044124866	4.682746733	0.000113886	0.064029361
cg18009653	Down	4	10076546	WDR1	3'UTR	-306.6680454675	56.7372669584	-5.4050549473	1.98720920190834E-05	0.0638032515
cg00826638	Down	2	198541655	RFTN2	TSS1500	-266.9723563994	61.6464459106	-4.3307015101	0.0002689918	0.0633820366
cg00533923	Up	6	116892543	RWDD1	TSS200	258.003012083	45.6117398742	5.6565045051	1.09239487448583E-05	0.0633084976
cg25099065	Up	2	69994225	ANXA4	5'UTR	275.9385035546	56.226849309	4.9075932041	6.5911902672604E-05	0.0631675998
cg23908998	Up	10	22292698	DNAJC1	TSS200	345.6265592834	57.3334408466	6.0283589155	4.56169239029378E-06	0.0631449725
cg25628481	Up	11	19368112			301.3867637368	58.6198676665	5.1413757099	3.74362208551229E-05	0.0629397019
cg18110478	Down	8	974818			-330.657192544	74.6189217041	-4.4312780859	0.0002103815	0.0629264144
cg01763090	Up	15	31775406	OTUD7A	3'UTR	298.7361829789	69.0669895533	4.3253106138	0.0002725589	0.0624270915
cg14610217	Down	1	27334888	FAM46B	Body	-281.5507217293	61.5415621133	-4.5749687213	0.0001481232	0.0622605606
cg09000830	Up	1	94883752	ABCD3	TSS200	319.2373571555	53.7810930182	5.9358659194	5.66053779554038E-06	0.0620480269
cg02294055	Up	2	109746170	LOC100287216; SH3RF3	Body; 1stExon	263.9886442157	54.9272253172	4.8061529176	8.43316703339159E-05	0.0619051678
cg01802258	Up	14	92790097	SLC24A4	TSS200; 5'UTR	287.7904837844	59.5766297466	4.8305935567	7.94664126736131E-05	0.0618608843
cg11828669	Up	14	64971476	ZBTB1; ZBTB25	5'UTR; TSS1500; 1stExon	289.2320032356	59.5386575312	4.8578858716	0.000074367	0.0617415157
cg08700690	Up	15	60884630	RORA	Body; 5'UTR; 1stExon	364.6907715408	60.7725035509	6.0009173595	4.86286200468829E-06	0.0612897724
cg21652832	Up	17	16395644	C17orf76	TSS200	300.4149169754	55.9657850045	5.3678317378	2.17231718238114E-05	0.0610577991
cg01547742	Up	6	32821155	PSMB9; TAP1	TSS1500; 1stExon	324.6318601182	68.6946279517	4.7257241184	0.0001025649	0.0609474785
cg24155429	Up	3	185911885	DGKG	Body	266.3690079142	57.2676799257	4.6512973506	0.0001229598	0.060615472
cg26275848	Down	12	49355334			-259.1754876455	62.371015345	-4.1553834936	0.0004128223	0.0604778389
cg00265238	Up	7	39989474	CDK13	TSS1500	294.139239307	50.9181204001	5.7767104715	8.22413151972654E-06	0.0602542432
cg08497766	Up	10	6019877	IL15RA	TSS1500; 5'UTR; 1stExon	301.8057855526	52.3052238629	5.7700887839	8.3534318340805E-06	0.0601038408
cg20638429	Down	22	24979642	GGT1	TSS200	-320.8040603015	67.3690904547	-4.7618879539	9.39206141222125E-05	0.0598666987
cg04528240	Down	4	56504657			-277.0428403217	62.8932089118	-4.4049722556	0.0002243469	0.0592320963
cg00379080	Up	12	15942595	EPS8	TSS200	277.1125709943	35.5198668133	7.801621905	8.93338549793308E-08	0.0589489618
cg22840780	Up	13	58207650	PCDH17	1stExon	275.6909104908	66.3856930537	4.1528663453	0.0004153677	0.0588693726
cg21995261	Down	11	4631906			-360.7718713287	82.1085621072	-4.3938398392	0.0002305331	0.0588077922
cg10399269	Down	16	1205342	CACNA1H	Body	-412.9643214666	74.052218059	-5.576663769	1.3201192171168E-05	0.058509929
cg08817120	Up	4	41363320	LIMCH1	Body	300.1499438289	62.0993935688	4.8333796287	7.89300832646128E-05	0.0584017754
cg16358867	Down	17	72306008	DNAI2	Body	-249.6546461358	57.7330462461	-4.3242936649	0.0002732371	0.0582642544
cg08877948	Down	16	58059154	MMP15	TSS200	-292.0488444788	76.3084723616	-3.8272138786	0.0009183675	0.0580451031
cg03273509	Up	18	9913806	VAPA	TSS200	341.249198806	44.3783089301	7.6895494	1.13155334863477E-07	0.0580384404

cg08023265	Up	1	31158138			363.9793157318	69.5050441378	5.2367323875	2.97543055533359E-05	0.0575926071
cg27395891	Up	20	34893942			327.6245140226	76.6956673104	4.2717473556	0.0003106744	0.0564723296
cg04201253	Up	2	177022517			322.0966638146	51.1224440403	6.3004942322	2.43112196195613E-06	0.0558914294
cg02921269	Up	14	75894323	JDP2	TSS200; TSS1500	340.0143409214	50.7654204848	6.6977548432	9.8577948193839E-07	0.0554980115
cg17265120	Down	17	42987382	GFAP	Body; 3'UTR	-309.4242720801	62.5411663598	-4.9475296047	5.98264397395277E-05	0.055495235
cg11019008	Up	10	131425282	MGMT	Body	319.4956277879	69.0698001867	4.6256920814	0.000130882	0.0553674072
cg16222848	Up	13	42846088	AKAP11	TSS200	360.5611941717	75.7091335481	4.7624530525	9.37915266933907E-05	0.0551651635
cg13896157	Up	1	27286832	C1orf172	5'UTR	398.5022549283	77.0937892078	5.1690578323	3.50192401195276E-05	0.0544854693
cg06467910	Up	11	33037542	DEPDC7	1stExon; TSS200	296.6965803531	57.9517167162	5.1197202976	3.94442242583974E-05	0.0544783142
cg12681001	Down	6	31543540	TNF	1stExon	-288.3907590279	64.5963953261	-4.4645023545	0.0001939809	0.0544137253
cg23784675	Down	18	34837596	BRUNOL4	Body	-385.9425545839	62.2981669187	-6.1950868488	0.000003099	0.0541666841
cg03294619	Up	5	172661803	NKX2-5	1stExon	411.4165568075	87.3055898548	4.7123736005	0.0001059548	0.0536169973
cg05847784	Up	12	108237749			369.3596522392	68.4042503668	5.3996593817	2.01301523365393E-05	0.0535165156
cg12071544	Up	16	4422228	VASN; CORO7	5'UTR; Body	340.0620578465	67.4409530664	5.0423673211	4.75500592170456E-05	0.0533337594
cg19026573	Up	12	133137360	FBRSL1	Body	331.0543796285	63.4483865304	5.2176957955	3.1148582889388E-05	0.0533330788
cg23646044	Down	17	42941002	EFTUD2	Body	-376.0206969682	69.2184200957	-5.4323790755	1.86158770840967E-05	0.053121772
cg15417285	Up	11	124733557			322.1982661056	64.2744633616	5.0128503492	5.10703284923632E-05	0.0530613757
cg01105963	Up	14	103589413			368.6565359543	55.4167393348	6.6524400457	1.09160419156592E-06	0.0530260563
cg13948413	Up	1	236687580	LGALS8	5'UTR	337.1839398651	76.216916678	4.4240039425	0.0002141537	0.0528974337
cg21273013	Up	1	23670943	HNRNPR	TSS200	332.7574101504	49.7023262136	6.6950067633	9.91887006060749E-07	0.0528627685
cg20942223	Up	7	155251040	EN2	1stExon; 5'UTR	438.0755755644	77.9951815258	5.6167005063	1.20044691209003E-05	0.052828339
cg00477061	Up	18	77794585	C18orf22	1stExon	377.7420300311	60.4452421653	6.249326109	2.7347014715191E-06	0.0524637684
cg26468336	Up	3	55523173			345.1241432727	76.3185677929	4.5221517286	0.0001685059	0.0517674607
cg08703950	Up	15	82338222	MEX3B	1stExon; 5'UTR	382.0481459432	66.871449335	5.7131728076	9.55385071673003E-06	0.0515703369
cg21182903	Up	20	56284556	PMEPA1	5'UTR; 1stExon; Body	444.1813892691	73.0503000866	6.0804868528	4.04094910604123E-06	0.0512089347
cg04613791	Up	20	1373260	FKBP1A	Body	339.0128930744	70.8114263049	4.7875450441	8.82362550473989E-05	0.0509922052
cg03996195	Up	18	55095229			369.788766872	73.2748957878	5.0465956027	4.70662720741391E-05	0.0507623846
cg04488845	Up	11	19262618	E2F8	TSS200	369.7743013799	67.0012288827	5.5189181982	1.51444743675916E-05	0.0503429175
cg07849904	Up	22	28197796	MN1	TSS1500	380.4501194818	71.5090345302	5.3203084335	2.43433275425001E-05	0.0499009637
cg05396987	Up	4	122686269	TMEM155; LOC100192379	1stExon; 5'UTR; Body	475.2779963452	88.5338659273	5.3683185679	2.16978662180124E-05	0.0497548947
cg17419175	Up	2	133037546			388.890014164	81.0046650469	4.8008347907	8.54294838059426E-05	0.0497525403
cg21343919	Up	20	46413743	SULF2	5'UTR	427.1003322739	63.0063789126	6.7786839943	8.22189310616406E-07	0.0495374894
cg07756148	Up	19	14247627	ASF1B	TSS200	365.9512677716	78.2917637949	4.6741987922	0.0001162834	0.0495178627
cg19572135	Down	X	23925992	APOO; CXorf58	1stExon; 5'UTR; TSS200; Body	-406.893699845	77.9475597953	-5.2200954195	3.09692454033011E-05	0.0490123149
cg15936446	Up	5	42952369			339.7670860988	70.5656988833	4.8149042874	0.000082556	0.0488152312
cg24455205	Up	20	18118988	PET117	Body	486.3839250789	80.5493313726	6.0383359711	0.000004457	0.0484216948
cg10059378	Up	16	4817345	ZNF500	TSS200	443.5113349292	78.249420249	5.6679184781	1.06327532693931E-05	0.0483494708
cg18128212	Down	1	26199190	PAQR7	TSS1500	-341.1792480602	74.114795653	-4.6033891756	0.0001381996	0.0478952147
cg26896160	Up	1	6454004	ACOT7	TSS200	367.0616942012	75.6473694382	4.8522730787	7.53880564641755E-05	0.0478384952
cg09374375	Up	6	32821161	PSMB9; TAP1	TSS1500; 1stExon	413.4617689521	74.258296965	5.5678864969	1.34793746101553E-05	0.0476620287
cg01182555	Up	14	62162064	HIF1A	TSS200	401.2761208411	59.0625200157	6.7940907488	7.94346868772002E-07	0.0475920562
cg26228542	Up	11	43965778			384.4532046798	81.1766268063	4.7360086247	0.0001000281	0.047342409

cg26800884	Up	4	39448008	KLB	Body	475.4756026401	70.8913891921	6.7070995231	9.65298141816118E-07	0.0472297265
cg01477633	Up	10	71078714	HK1	Body; 1stExon	390.3713638154	73.6172907309	5.3027129896	2.53928702654396E-05	0.0471030628
cg01296018	Up	2	207668519			454.7810900519	87.8107021092	5.1791077753	3.41813843088234E-05	0.0466588787
cg14124917	Up	2	10090974	GRHL1	TSS1500	323.3021391945	76.8077985224	4.2092358512	0.0003619406	0.046591459
cg12016437	Up	4	10459163	ZNF518B	TSS200	392.0285189206	80.58996801	4.8644828705	7.31847442098869E-05	0.0465367143
cg06378770	Up	6	29521714			386.5757091265	67.9936643001	5.6854666255	1.02003874129639E-05	0.0462005805
cg25097801	Up	8	23315147	ENTPD4	TSS200; 1stExon; 5'UTR	446.4100263741	74.7422706956	5.9726580718	5.1942571416839E-06	0.0459982628
cg13636189	Up	9	102587074	NR4A3	5'UTR	463.6096036418	67.2745895037	6.8913033444	6.39606301889721E-07	0.0458026024
cg06482019	Up	16	51147902			392.5414949757	87.2156649721	4.5008141038	0.0001775183	0.0455812949
cg02164386	Down	17	42925536	HIGD1B	1stExon	-400.1571052705	98.77563168	-4.0511723232	0.0005323903	0.0454845497
cg01089095	Up	10	75541668	CHCHD1	TSS200	417.7617084155	66.4709579513	6.2848757005	2.51994504306368E-06	0.0454501682
cg21913376	Down	2	55366440			-413.0235454934	82.1891735735	-5.025279213	0.000049557	0.0450611248
cg22978087	Up	8	24814126	NEFL	5'UTR; 1stExon	388.0317015445	92.8571643061	4.1788019745	0.0003898735	0.044824766
cg12548227	Up	7	6144298	USP42	TSS1500	390.5104218822	70.546145088	5.535531692	1.45573214731769E-05	0.044545233
ch.2.12395676F	Down	2	12478225			-366.7365871365	87.1599760842	-4.2076260643	0.0003633668	0.0445447511
cg01532694	Up	8	8243576			399.5960752127	70.7043307319	5.6516492141	1.10502661869462E-05	0.0444505124
cg19547629	Up	3	39851641	MYRIP	5'UTR	480.1904586233	93.6561212076	5.127165768	3.87418303277378E-05	0.0437790252
cg20923716	Up	18	32557027	MAPRE2	5'UTR; 1stExon; TSS1500	431.0431930386	81.6783571156	5.2773244744	2.69887056488158E-05	0.0437047259
cg02251742	Up	3	156009319	KCNAB1	Body; 1stExon; 5'UTR	373.9272154451	75.4055653559	4.9588808688	5.82026108378368E-05	0.0432354899
cg13911723	Up	7	6655120	ZNF853	TSS1500	518.3896428098	85.0161777736	6.0975411549	3.88409485438474E-06	0.0428027687
cg20771178	Up	11	8615675	STK33	TSS200	386.399865869	89.2986473135	4.3270517247	0.0002714017	0.0425218459
cg26758863	Up	19	57752111	ZNF805	1stExon; 5'UTR	463.0948391786	89.2035136992	5.1914416818	3.31808014243971E-05	0.0424568265
cg17682704	Up	4	75023746	MTHFD2L	TSS200	423.5273227586	90.2529688624	4.6926691509	0.0001111653	0.0424405678
cg00123094	Up	16	1662131	IFT140	TSS200	403.7269998783	69.005797683	5.8506243451	0.000006912	0.0420528354
cg07017477	Up	1	151882157	THEM4	TSS200	441.21035685	91.9234269545	4.7997596638	8.56531650087655E-05	0.0420020435
cg03393426	Up	10	102986686			425.9291589288	98.1768532019	4.3383867484	0.0002639871	0.0419282597
cg10517474	Up	16	31470676	ARMC5	5'UTR; 1stExon	447.828202921	86.3650953004	5.1852915968	0.000033676	0.0418614037
cg03506372	Down	11	60897603			-404.342641497	97.8580200992	-4.1319315585	0.0004371528	0.0414995055
cg25635316	Up	7	23637023	CCDC126	5'UTR; 1stExon	433.6653468557	76.4887944719	5.6696585408	1.05890595539216E-05	0.0413504984
cg26676559	Up	11	14995167	CALCA	TSS1500	414.9040553926	85.4468097558	4.8556997807	0.000074763	0.0413180134
cg21316470	Up	15	63340581	TPM1	Body; TSS200	419.0038988753	65.7779310087	6.3699768669	2.07314988491689E-06	0.0412032436
cg16134139	Down	7	20042353			-428.5813564911	105.6221874737	-4.0576830185	0.0005240014	0.0408366418
cg15901997	Up	18	48723744	MEX3C	1stExon; 5'UTR	367.5399175936	65.4312055574	5.6171961752	1.19903660075247E-05	0.0407345609
cg03816593	Up	20	30311703	BCL2L1	TSS1500	479.9067218061	101.815884614	4.7134759338	0.0001056707	0.0406581406
cg23922708	Up	18	78005180	PARD6G	1stExon	473.2677806216	88.9671898949	5.3195765898	2.43860913576969E-05	0.0405980029
cg26411702	Up	8	21881998	NPM2	TSS1500	462.1338786737	81.1044246729	5.6980106885	9.90232139344012E-06	0.0399547905
cg17731560	Down	15	93152765			-402.1735899197	92.0204741384	-4.3704794361	0.0002440752	0.0399145986
cg14464791	Down	11	118661599	DDX6	5'UTR	-359.5602399454	104.4554047322	-3.4422368174	0.0023244908	0.0396418521
cg07819926	Up	7	139025145	C7orf55	TSS1500	425.3299708885	74.0961540268	5.7402435589	8.96241475516333E-06	0.039612374
cg09243852	Down	7	1160383	C7orf50	Body	-480.449094151	100.526587526	-4.7793236195	9.00189444127709E-05	0.0394559776
cg02564756	Up	3	128902977	CNBP	TSS200	465.7101894313	93.526796536	4.9794305662	5.53752842787346E-05	0.0392752962
cg01886303	Down	15	29406026	APBA2	Body	-487.6939212583	113.3995329713	-4.3006695749	0.0002894754	0.0389968568

cg18469326	Up	17	29158893	ATAD5	TSS200	519.2741050743	100.3578196935	5.1742266488	3.45857541381866E-05	0.0388451719
cg02442412	Up	4	83720054	SCD5	TSS200	474.2148187994	90.0246410086	5.2676113283	2.76258071917186E-05	0.0387528565
cg07249227	Up	21	43430503	ZNF295	TSS200	450.0456974167	79.9921835283	5.6261209229	1.17392970152843E-05	0.0383800276
cg19720914	Up	1	234746494	IRF2BP2	TSS1500	488.1524203677	94.7671179838	5.1510738192	3.65708130464232E-05	0.0383045298
cg03371770	Up	2	96932024	CIAO1; TMEM127	1stExon; 5'UTR; TSS1500	506.2040887189	98.7380861768	5.1267358759	0.000038782	0.0382367284
cg13379794	Up	5	3590590			518.8545664527	96.2354573068	5.3915114135	2.05263185565984E-05	0.0378651699
cg23273214	Up	19	14142737	IL27RA	1stExon	519.017571808	88.3727341041	5.8730509706	6.55762433711899E-06	0.0375494093
cg02019051	Up	15	49913146	DTWD1; C15orf33	TSS200	527.4596437966	101.8046155536	5.1810975458	3.40179213872748E-05	0.0370872101
cg00816008	Up	16	82203996	MPHOSPH6	TSS200	415.1390853368	64.3315260639	6.4531204331	1.71470252084757E-06	0.0370687087
cg18238491	Up	18	47901440	SKA1	5'UTR; 1stExon	506.8010465317	86.6946432615	5.8458173131	6.99041818327729E-06	0.0369013301
cg18565702	Up	13	113241891	TUBGCP3	Body	395.6393564134	69.3283649662	5.7067458119	0.0000097	0.0368456578
cg04971418	Up	5	93954596	C5orf36; ANKRD32	TSS1500; 5'UTR	453.6069746291	71.3427940069	6.3581330244	2.13012149881327E-06	0.0367521262
cg22982536	Up	13	24735537	SPATA13; MIR2276	5'UTR; TSS1500	528.3576773077	93.6931735644	5.6392334383	1.13800839599895E-05	0.0365974366
cg10298855	Up	14	35874025	NFKBIA	TSS200	513.6773310431	96.5110454324	5.3224719382	2.42173517599905E-05	0.0365767006
cg04541154	Up	5	177026954	B4GALT7	TSS200	506.0199099007	127.2408148465	3.9768678825	0.0006381212	0.0361931977
cg07377675	Up	1	62901875	USP1	TSS200; TSS1500	536.9539611599	94.9398117594	5.6557302064	0.000010944	0.0358391787
cg06191357	Down	1	172714273			-497.947112215	98.4123654462	-5.0598022917	0.000045587	0.0357305469
ch.3.638689R	Down	3	31240746			-528.5425854707	122.7190654208	-4.3069313122	0.0002850796	0.0355381457
cg17148467	Up	2	242641476	ING5	1stExon; 5'UTR	484.1595930739	91.2129055544	5.3080163397	2.50717894961033E-05	0.0355012288
cg07161071	Up	7	128578164	IRF5	TSS200; 5'UTR	478.2732409885	85.1556774152	5.6164574754	1.20113901686976E-05	0.0354776399
ch.5.2235120R	Down	5	122721651	CEP120	Body	-380.4624516979	95.6438133705	-3.9779096869	0.0006365035	0.0350915133
cg19287139	Up	10	75634631	CAMK2G	TSS1500	447.1421127664	105.2994437068	4.2463862773	0.0003305357	0.0350297093
cg23811775	Up	19	4402960	CHAF1A	Body	575.6489890408	106.9650045794	5.3816572187	0.000021016	0.0341353272
cg20189674	Up	17	35295051	LHX1	1stExon; 5'UTR	493.2267408318	85.543035524	5.7658316403	8.4376520169636E-06	0.0340038304
cg26670485	Up	1	244615549	ADSS	TSS200	550.8456278917	111.5200950619	4.9394293251	6.10131109500376E-05	0.0339851591
cg18867283	Up	1	94374612	GCLM	1stExon	476.5073629562	105.9815627295	4.4961345227	0.0001795586	0.0338412169
cg17386700	Up	16	88729471	MGC23284; MVD	TSS1500; 1stExon	620.4188031551	86.6816928415	7.157437549	3.55666672128424E-07	0.0337845248
cg25978090	Up	4	2011084	WHSC2	TSS200	562.5710121588	109.5156676805	5.1368998069	3.78425900598839E-05	0.0337727148
cg02926622	Down	6	144386041	PLAGL1	TSS1500	-668.6133572873	120.6763529323	-5.5405499175	1.43845579701569E-05	0.0333590451
cg24184687	Up	10	1779760	ADARB2	TSS200	547.6808724803	104.8471558269	5.2236121062	3.07083115754563E-05	0.032596893
cg01366595	Up	5	42952307			611.7800995503	150.7631561334	4.0578886463	0.0005237387	0.0321972867
cg20458124	Up	1	25869988	LDLRAP1	TSS200	597.5135074584	122.9876032569	4.8583230475	7.42880729249769E-05	0.0316215998
cg00528464	Up	1	52498993	TXNDC12; KTI12	Body; 1stExon	565.9144763481	110.0337426528	5.1430994048	3.72809064238613E-05	0.0316188772
cg15486822	Up	6	34360745	NUDT3	TSS1500	668.6910549758	124.2215829376	5.3830505067	2.09460805554492E-05	0.0315655825
cg04258457	Up	17	62207789	ERN1	TSS1500	480.1473173242	87.3826631391	5.4947663538	1.60412717114094E-05	0.0314249847
cg22640847	Up	7	150945607	SMARCD3	Body; 1stExon	667.0307814403	124.397915566	5.36207362	2.20247627619073E-05	0.031279674
cg00795915	Up	5	70751728	BDP1	1stExon	577.7140890262	99.4452931779	5.8093658389	7.61567068188432E-06	0.031232993
cg14452096	Up	9	127703551	GOLGA1	TSS200	582.8327705631	122.7823511272	4.7468774234	9.74158129429991E-05	0.0310637291
cg06779945	Up	6	138188320	TNFAIP3	TSS1500	526.7273850262	96.1599520312	5.4776169694	1.67106579665496E-05	0.0310605039
cg06519449	Up	7	97601680	MGC72080	TSS200	699.3536080961	126.4364815137	5.5312643924	1.4705891348003E-05	0.0300483539
cg15233292	Up	22	32340329	YWHAH; C22orf24	TSS200; 5'UTR	697.263158357	134.1361759842	5.1981738203	3.26472272216657E-05	0.0294678051
cg12921992	Up	7	44887755	H2AFV	TSS200	555.0541203915	96.6057225967	5.7455614996	8.85068341172909E-06	0.0294142117

cg10492801	Up	17	59476859	TBX2	TSS1500	611.8084074337	114.5449602295	5.3412075591	2.31537051273105E-05	0.0293223323
cg03607359	Up	5	176057465	SNCB; EIF4E1B	5'UTR; 1stExon; TSS1500	555.123848339	83.483446979	6.6495079974	1.09884018780052E-06	0.0285384922
cg07042827	Up	14	90849692			607.9974523087	126.2048118539	4.8175457289	8.20274841475923E-05	0.0279208486
cg22848779	Up	4	103997452	NHEDC2	1stExon; 5'UTR	762.469126376	138.4003864258	5.5091546062	1.55007115864919E-05	0.0277262884
cg25966904	Up	6	32862630			621.8760042077	130.8654484794	4.7520259277	9.62024648824337E-05	0.026716392
cg08538929	Up	21	35013827	ITSN1; CRYZL1	TSS1500; 5'UTR	609.7385594961	127.5174484912	4.7816088442	8.9519817976099E-05	0.0266385917
ch.11.1877857R	Down	11	88218727			-652.7196369607	163.6095076875	-3.9894969809	0.0006187827	0.0263680258
cg02650728	Down	4	2942362	C4orf10; NOP14	Body	-620.4344225723	123.0979655817	-5.0401679641	4.78036876049924E-05	0.0262039687
cg21317795	Up	6	138725205	HEBP2	TSS200	755.8460109783	162.8957058013	4.6400609965	0.0001263752	0.0260216477
cg11992317	Up	20	44520160	CTSA; NEURL2	5'UTR; Body; TSS1500	765.2619302786	149.860118119	5.1065082551	4.07225097976943E-05	0.025685171
cg19199531	Up	22	20104664	TRMT2A; RANBP1	1stExon; TSS1500; 5'UTR	706.0032371081	135.4462051179	5.2124253795	3.15461731323787E-05	0.0256748325
cg08936794	Up	1	43833873	ELOVL1	TSS200	648.5817779303	110.8232195269	5.852399711	6.88321296475195E-06	0.0254339717
cg01617425	Up	3	47844262	DHX30	TSS200	743.5206127668	143.8535931724	5.1685925695	3.5058528566058E-05	0.0248016385
cg06958537	Down	21	40176950	ETS2	TSS1500	-544.8118426429	136.6779685224	-3.9860984805	0.0006239288	0.024714698
cg02382073	Up	4	106067967	TET2	5'UTR; 1stExon	681.0337235938	128.9610138183	5.2809271843	2.67562026507898E-05	0.0244484478
cg06439982	Up	16	2802372	SRRM2; LOC100128788	5'UTR; Body; 1stExon	866.5067571303	116.9887664214	7.4067518073	0.000000207	0.0238343004
cg11365324	Up	5	98265264			826.1590233713	143.1212303439	5.7724421554	8.30724249935189E-06	0.0228719996
cg11044099	Up	8	102092965			810.8826699051	188.3441023217	4.3053255181	0.0002862005	0.0227282704
cg12940104	Up	6	108439511			797.3572068085	161.9925449095	4.9221845811	6.36191952652746E-05	0.0220110881
cg18118497	Up	5	66299875	MAST4	Body	903.4896574726	174.5093647384	5.177313314	3.43294843540729E-05	0.0203285268
cg07757096	Down	5	77591498	AP3B1	TSS1500	-859.7682255008	217.9059878649	-3.9455924728	0.0006886331	0.0191939991
cg05191824	Up	12	123868698	SETD8	TSS200	878.6656649394	191.012797669	4.600035577	0.0001393349	0.0189898649
cg21639168	Up	11	10562708	RNF141	1stExon; 5'UTR	1027.8567027755	243.1812543802	4.2267102594	0.0003468141	0.017584008
cg09691810	Up	5	34915636	RAD1; BRIX1	1stExon; Body; TSS200; 5'UTR	989.7917893956	249.9903402185	3.9593201423	0.0006659911	0.0169255499
cg07353572	Up	1	91966518	CDC7	TSS200; 5'UTR	1260.4898431602	155.6982430704	8.0957229722	4.84273389012159E-08	0.0168309061
cg09758595	Up	8	11141996	MTMR9	TSS200	1042.6758830903	258.5469078434	4.0328306062	0.0005567478	0.0159920551
cg20105384	Up	13	73301976	C13orf37; C13orf34	TSS200	1321.4941799197	250.5369449303	5.2746479378	2.71627627721732E-05	0.0155642023
cg06398166	Up	3	49760915	GMPPB	Body	1106.171834494	172.9141784595	6.3972303738	1.9479005242931E-06	0.015150644
cg07111501	Up	17	62207504	ERN1	TSS200	1094.2771842783	194.1731198035	5.6355750239	1.14791653876006E-05	0.0138817481
cg24678700	Up	5	76011138	F2R	TSS1500	1344.2679458453	211.0002366516	6.3709309865	2.0686287518329E-06	0.0134324053
cg26094983	Up	17	65242096	HELZ	TSS1500	1721.7806699891	294.4051266916	5.8483379326	6.94916607647434E-06	0.0132927584
cg08663092	Up	14	74181037	PNMA1	1stExon; 5'UTR	1553.1940749199	235.5611409921	6.5935920856	1.24665075832673E-06	0.0131378477
cg14022137	Up	6	150285634	ULBP1	Body	1569.3496352528	338.4069431283	4.6374628746	0.0001271784	0.0123784933
cg00024472	Up	17	47573864	NGFR	Body	1362.2598473589	286.3991236971	4.756508434	9.51584723398688E-05	0.0111408119
cg16699331	Up	5	110074725	SLC25A46	TSS200	1662.6775397226	342.6391708066	4.8525611821	7.53353039386763E-05	0.0107785467
cg08515845	Up	7	150755180	CDK5; SLC4A2	TSS200; TSS1500	1628.0902852922	331.5337120066	4.9107835081	6.54035959649942E-05	0.0097989403
