# Supplementary material for: Methylome-wide association study of whole blood DNA in the Norfolk Island isolate identifies robust loci associated with age
Source: Aging (Albany NY). 2017 Feb 28;9(3):753–66. doi: 10.18632/aging.101187 (PMC5391229; doi:10.18632/aging.101187)
Supplement: Supplementary file 3 [file aging-09-753-s003.pdf]

Table_S2_aging_toppgene_biological_process

Category	ID	Name	p-value	q-value Bonferroni	q-value FDR B&H	Hit Count in Query List	Hit Count in Genome
GO: Biological Process	GO:0045935	positive regulation of nucleobase-containing compound metabolic process	1.247E-07	0.000647	0.0003081	68	1832
GO: Biological Process	GO:0051254	positive regulation of RNA metabolic process	2.187E-07	0.001135	0.0003081	61	1593
GO: Biological Process	GO:1903508	positive regulation of nucleic acid-templated transcription	2.711E-07	0.001407	0.0003081	59	1528
GO: Biological Process	GO:0045893	positive regulation of transcription, DNA-templated	2.711E-07	0.001407	0.0003081	59	1528
GO: Biological Process	GO:1902680	positive regulation of RNA biosynthetic process	4.014E-07	0.002083	0.0003081	59	1546
GO: Biological Process	GO:0045595	regulation of cell differentiation	4.142E-07	0.002149	0.0003081	63	1699
GO: Biological Process	GO:0051173	positive regulation of nitrogen compound metabolic process	5.302E-07	0.002751	0.0003081	69	1944
GO: Biological Process	GO:0042692	muscle cell differentiation	5.605E-07	0.002909	0.0003081	25	412
GO: Biological Process	GO:0061061	muscle structure development	6.706E-07	0.00348	0.0003081	33	657
GO: Biological Process	GO:0009891	positive regulation of biosynthetic process	7.026E-07	0.003646	0.0003081	69	1959
GO: Biological Process	GO:0031328	positive regulation of cellular biosynthetic process	7.205E-07	0.003739	0.0003081	68	1921
GO: Biological Process	GO:0008284	positive regulation of cell proliferation	7.409E-07	0.003845	0.0003081	41	924
GO: Biological Process	GO:0009790	embryo development	7.718E-07	0.004005	0.0003081	47	1135
GO: Biological Process	GO:0010557	positive regulation of macromolecule biosynthetic process	9.095E-07	0.004719	0.0003371	64	1777
GO: Biological Process	GO:0022008	neurogenesis	0.000002158	0.0112	0.0007039	59	1628
GO: Biological Process	GO:0035336	long-chain fatty-acyl-CoA metabolic process	0.000002171	0.01126	0.0007039	8	45
GO: Biological Process	GO:1901215	negative regulation of neuron death	0.000002556	0.01327	0.0007803	16	204
GO: Biological Process	GO:0048646	anatomical structure formation involved in morphogenesis	0.000002751	0.01428	0.0007932	50	1299
GO: Biological Process	GO:0051960	regulation of nervous system development	0.000003783	0.01963	0.000997	38	881
GO: Biological Process	GO:0045944	positive regulation of transcription from RNA polymerase II promoter	0.000003843	0.01994	0.000997	44	1094
GO: Biological Process	GO:0050767	regulation of neurogenesis	0.000004429	0.02298	0.001094	35	784
GO: Biological Process	GO:0035337	fatty-acyl-CoA metabolic process	0.000005793	0.03006	0.001334	8	51
GO: Biological Process	GO:0045596	negative regulation of cell differentiation	0.000006025	0.03126	0.001334	32	694
GO: Biological Process	GO:0048699	generation of neurons	0.000006169	0.03201	0.001334	55	1528
GO: Biological Process	GO:0010628	positive regulation of gene expression	0.000007105	0.03687	0.001475	64	1890
GO: Biological Process	GO:2000026	regulation of multicellular organismal development	0.000007478	0.0388	0.001492	64	1893
GO: Biological Process	GO:0060284	regulation of cell development	0.000008765	0.04548	0.001684	40	985
GO: Biological Process	GO:1901214	regulation of neuron death	0.000009862	0.05118	0.001769	19	307
GO: Biological Process	GO:0040023	establishment of nucleus localization	0.000009885	0.05129	0.001769	5	16
GO: Biological Process	GO:0045597	positive regulation of cell differentiation	0.00001307	0.06782	0.002261	39	966
GO: Biological Process	GO:0051647	nucleus localization	0.00001358	0.07046	0.002273	6	28
GO: Biological Process	GO:0043524	negative regulation of neuron apoptotic process	0.00001429	0.07416	0.002317	13	159
GO: Biological Process	GO:0051402	neuron apoptotic process	0.00001501	0.07788	0.00236	17	261
GO: Biological Process	GO:0070997	neuron death	0.00001571	0.08151	0.002397	20	346
GO: Biological Process	GO:0043523	regulation of neuron apoptotic process	0.00001631	0.08463	0.002418	16	236
GO: Biological Process	GO:0042127	regulation of cell proliferation	0.0000183	0.09498	0.002638	57	1666
GO: Biological Process	GO:0006357	regulation of transcription from RNA polymerase II promoter	0.00002126	0.1103	0.002982	63	1916
GO: Biological Process	GO:0007417	central nervous system development	0.00002966	0.1539	0.00405	39	1002
GO: Biological Process	GO:0009719	response to endogenous stimulus	0.00003247	0.1685	0.004321	58	1740
GO: Biological Process	GO:0043066	negative regulation of apoptotic process	0.00003902	0.2025	0.005062	36	905
GO: Biological Process	GO:0030182	neuron differentiation	0.00004176	0.2167	0.005286	49	1398
